# Supplementary material for: Coevolution of the olfactory organ and its receptor repertoire in ray-finned fishes
Source: BMC Biol. 2022 Sep 1;20:195. doi: 10.1186/s12915-022-01397-x (PMC9438307; doi:10.1186/s12915-022-01397-x)
Supplement: Supplementary file 1 — Additional file 1: Figure S1. Diversity of the olfactory receptor gene repertoire in ray-finned fishes. Figure S2. Distribution of ORA7 and ORA8 subfamilies in ray-finned fishes. Figure S3. Distribution of birth and death rates of OR, TAAR, OlfC and ORA genes in ray-finned fishes. Figure S4. Correlation of the number of gene losses (or gene gains) between gene families, estimated using the 368 branches of the phylogenetic tree. Figure S5. Correlation between the number of OR, TAAR and OlfC pseudogenes in 185 ray-finned fishes. Figure S6. Narial tubes of four species of ray-finned fishes with complex olfactory organs and large gene repertoires. Figure S7. Takifugu rubripes, USNM 57620, 290 mm TL. [file 12915_2022_1397_MOESM1_ESM.pdf]

Supplementary Figures

Fig. S1. Diversity of the olfactory receptor gene repertoire in ray-finned fishes. Time-calibrated phylogeny from https://fishtreeoflife.org/. (A) For each species, a multiple values barplot represents the number of functional genes, pseudogenes, truncated and edge genes in (A) ORA family, (B) OR family, (C) TAAR family, (D) OlfC family. When available, the olfactory epithelium shape and the number of lamellae is indicated. Inferred numbers of gene gains and losses are provided on branch of the trees. Whole-genome duplications are indicated by red stars. The branches associated with the three highest death rates and the two highest birth rates of the OR, TAAR and OlfC families are indicated by circles and diamonds, respectively, with color code as in Fig. 1. The trees were annotated and visualized using iTOL.

Fig. S2. Distribution of ORA7 and ORA8 subfamilies in ray-finned fishes. Time-calibrated phylogeny from https://fishtreeoflife.org/. (A) ORA7, (B) ORA8.

Fig. S3. Distribution of birth and death rates of OR, TAAR, OlfC and ORA genes in ray-finned fishes. $\bar{\beta}$: mean birth rate; $\bar{\delta}$: mean death rate. The two highest birth rates and the three highest death rates are indicated by colored arrows. Arrow colors correspond to the colors of circles and diamonds in Fig. 1 showing branches with high birth and death rates.

Fig. S4. Correlation of the number of gene losses (or gene gains) between gene families, estimated using the 368 branches of the phylogenetic tree. (A,D) Correlations between OR and TAAR families; (B,E) correlations between OR and OlfC families; (C,F) correlations between TAAR and OlfC families.

Fig. S5. Correlation between the number of OR, TAAR and OlfC pseudogenes in 185 ray-finned fishes. (A) Phylogenetic generalized linear regression (Pagel's λ model) between the number of OR and TAAR pseudogenes, (B) OR and OlfC pseudogenes, (C) TAAR and OlfC pseudogenes. (D) Phylogenetic generalized linear regression (Pagel's λ model) between the proportion of OR and TAAR pseudogenes, (E) OR and OlfC pseudogenes, (F) TAAR and OlfC pseudogenes. The coefficient of determination (R2), the p-value (P) and the regression line (solid line) of PGLS analyses are reported. Dotted line: slope = 1. Dot color code: as in Fig. 1.

Fig. S6. Narial tubes of four species of ray-finned fishes with complex olfactory organs and large gene repertoire. (A) *Erpetoichthys calabaricus*; photograph by Katherine E. Bemis, NOAA National Systematics Lab. (B) *Polypterus senegalus*, photograph by Basal Zoo. (C) Representative Anguillidae, *Anguilla japonica*, photograph by unknown. (D) *Mastacembelus armatus*, photograph by Zach Randall, Florida Museum of Natural History.

Fig. S7. *Takifugu rubripes*, USNM 57620, 290 mm TL. (A) Bridge of tissue between excurrent and incurrent nares. (B) Bridge of tissue cut and reflected to show lamellae in olfactory organ. Note that lamellae are not arranged in a rosette, but parallel to each other in a circle under the bridge and over the floor of the olfactory organ.

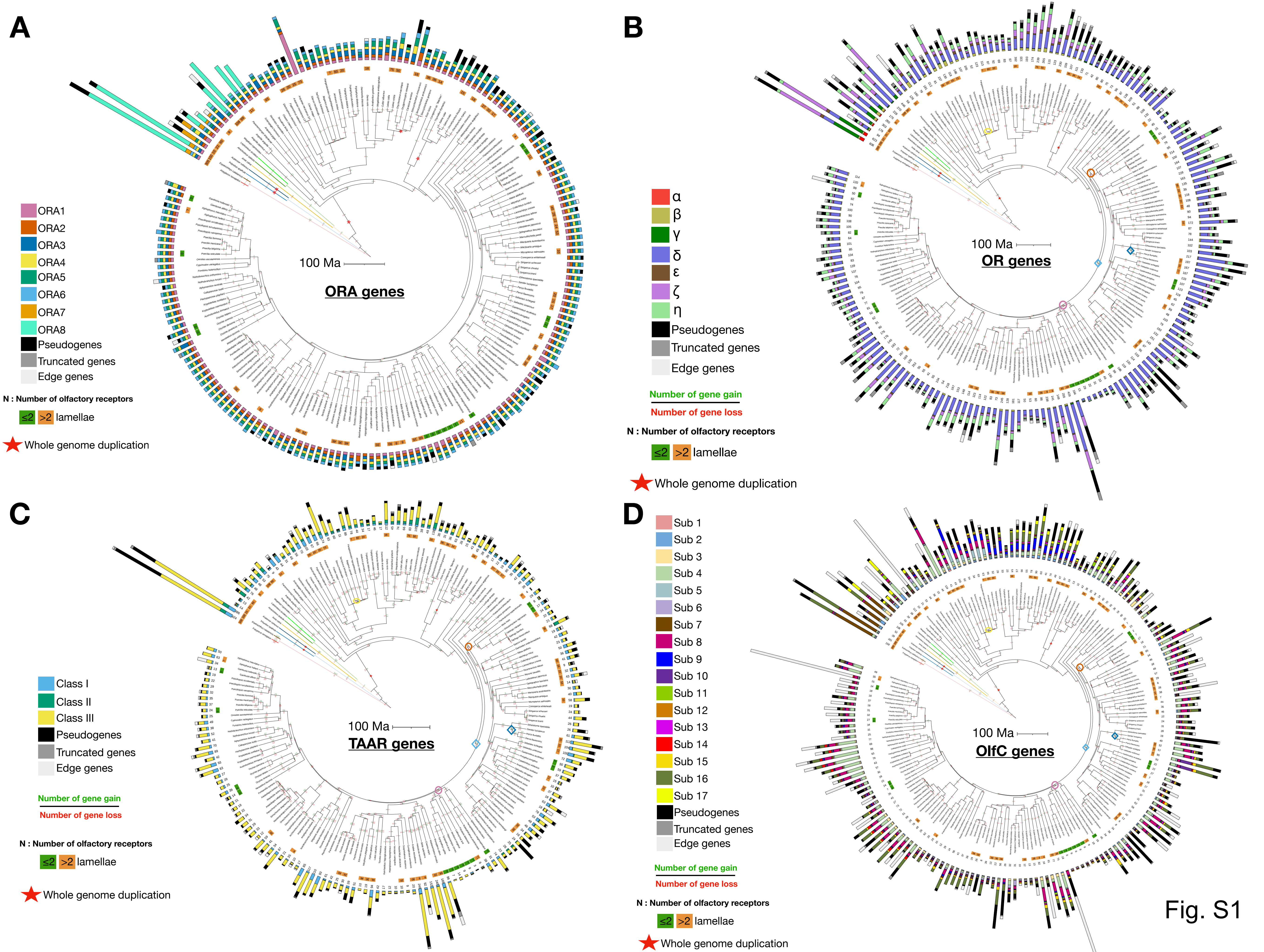
A
ORA1
ORA2
ORA3
ORA4
ORA5
ORA6
ORA7
ORA8
Pseudogenes
Truncated genes
Edge genes
N : Number of olfactory receptors
≤2 >2 lamellae
Whole genome duplication
100 Ma
ORA genes
B
α
β
γ
δ
ε
ζ
η
Pseudogenes
Truncated genes
Edge genes
Number of gene gain
Number of gene loss
N : Number of olfactory receptors
≤2 >2 lamellae
Whole genome duplication
100 Ma
OR genes
C
Class I
Class II
Class III
Pseudogenes
Truncated genes
Edge genes
Number of gene gain
Number of gene loss
N : Number of olfactory receptors
≤2 >2 lamellae
Whole genome duplication
100 Ma
TAAR genes
D
Sub 1
Sub 2
Sub 3
Sub 4
Sub 5
Sub 6
Sub 7
Sub 8
Sub 9
Sub 10
Sub 11
Sub 12
Sub 13
Sub 14
Sub 15
Sub 16
Sub 17
Pseudogenes
Truncated genes
Edge genes
Number of gene gain
Number of gene loss
N : Number of olfactory receptors
≤2 >2 lamellae
Whole genome duplication
100 Ma
OlfC genes

Fig. S1

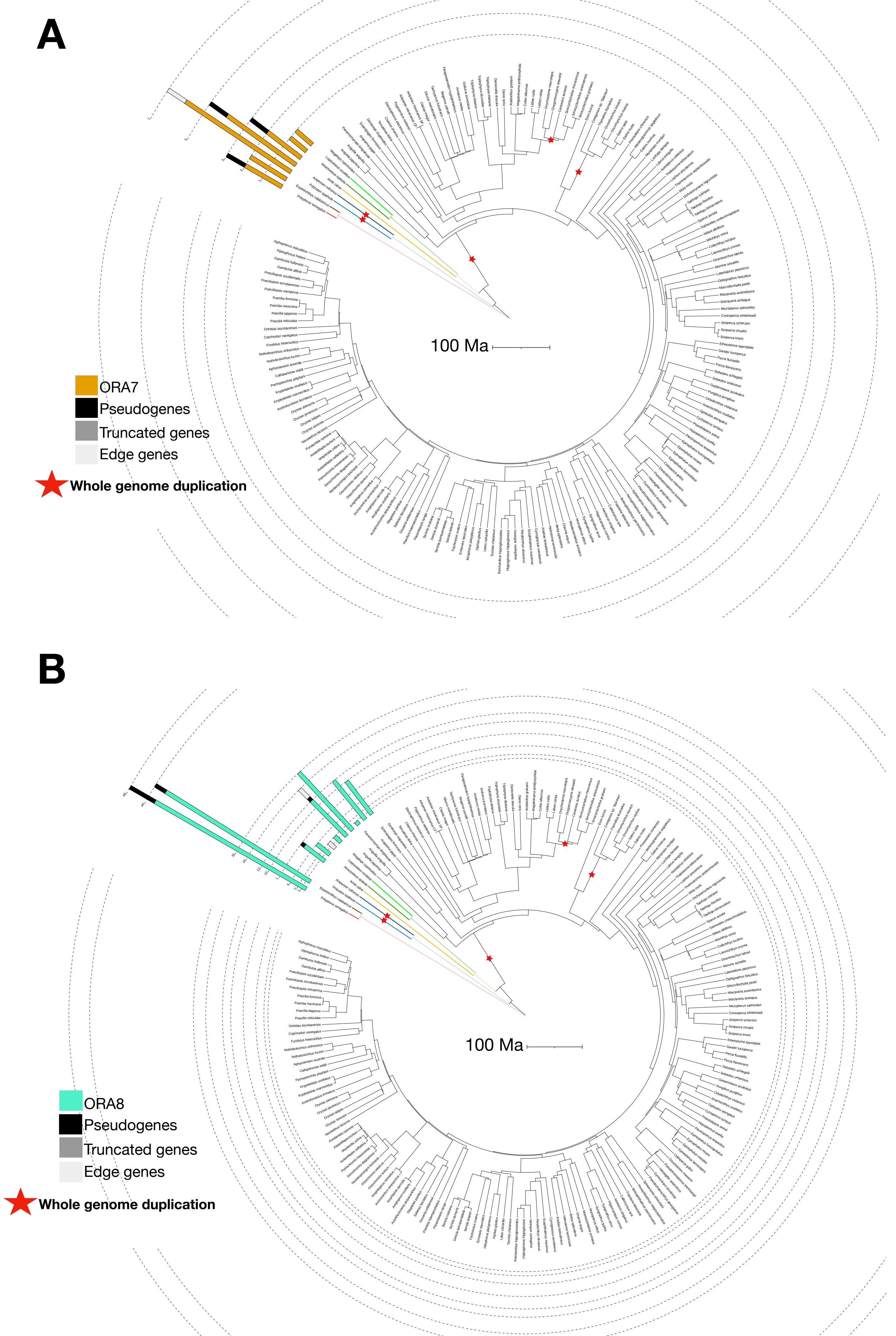
A
ORA7
Pseudogenes
Truncated genes
Edge genes
Whole genome duplication
100 Ma
B
ORA8
Pseudogenes
Truncated genes
Edge genes
Whole genome duplication
100 Ma

Fig. S2

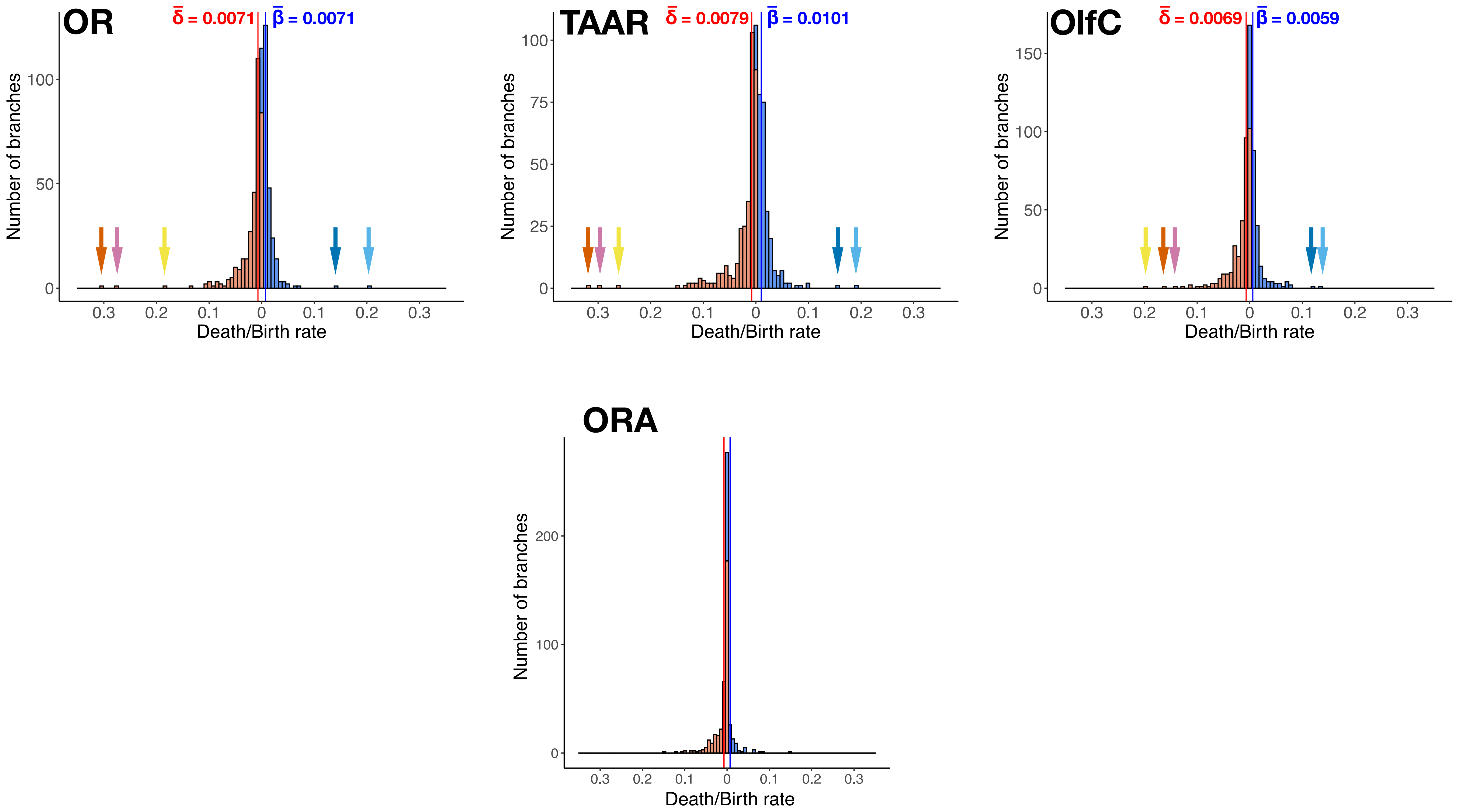
OR
δ̄ = 0.0071
β̄ = 0.0071
TAAR
δ̄ = 0.0079
β̄ = 0.0101
OlfC
δ̄ = 0.0069
β̄ = 0.0059
ORA
Number of branches
Death/Birth rate

Fig. S3

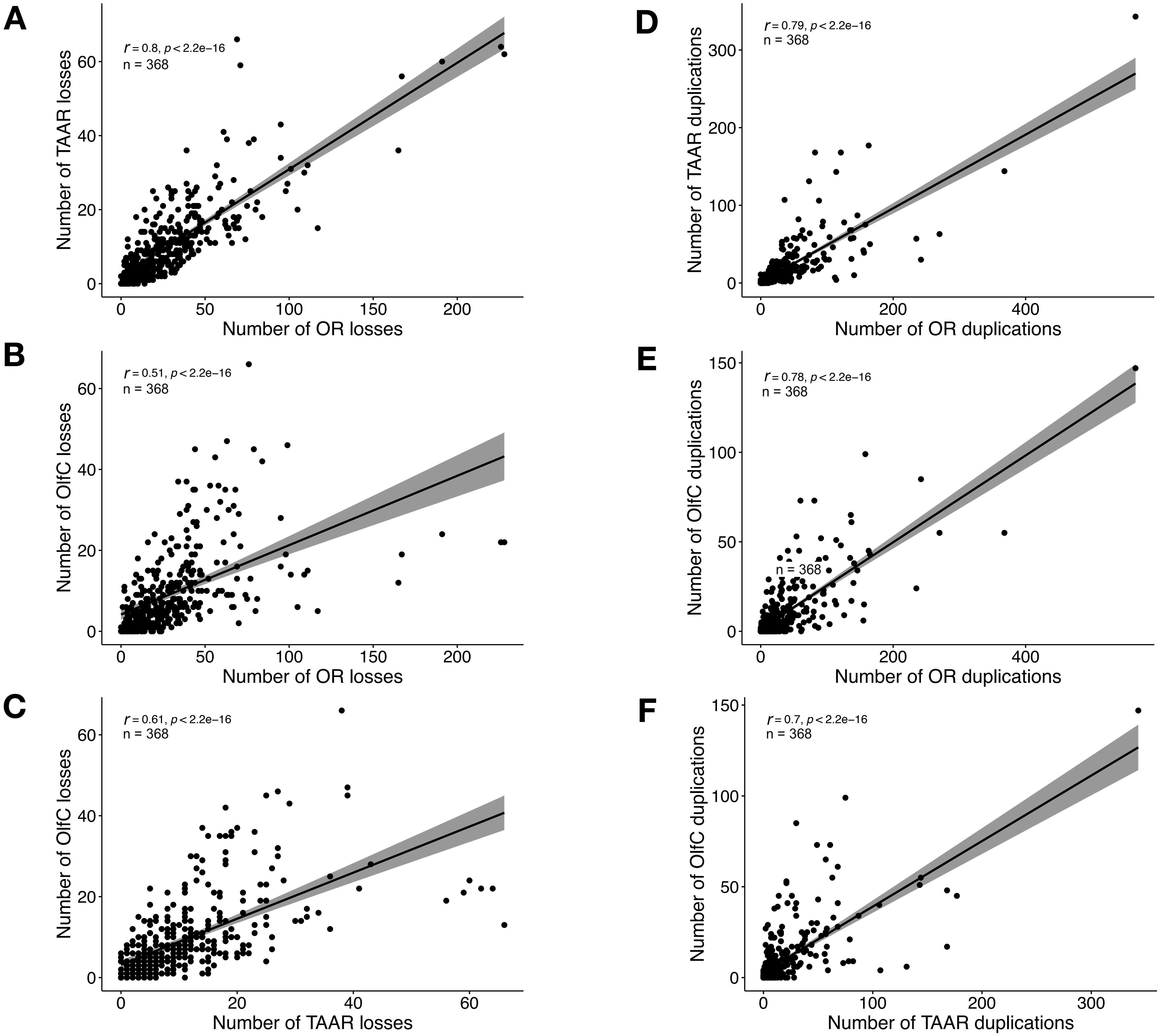
A
r = 0.8, p < 2.2e−16
n = 368
Number of TAAR losses
Number of OR losses
B
r = 0.51, p < 2.2e−16
n = 368
Number of OlfC losses
Number of OR losses
C
r = 0.61, p < 2.2e−16
n = 368
Number of OlfC losses
Number of TAAR losses
D
r = 0.79, p < 2.2e−16
n = 368
Number of TAAR duplications
Number of OR duplications
E
r = 0.78, p < 2.2e−16
n = 368
Number of OlfC duplications
Number of OR duplications
F
r = 0.7, p < 2.2e−16
n = 368
Number of OlfC duplications
Number of TAAR duplications

Fig. S4

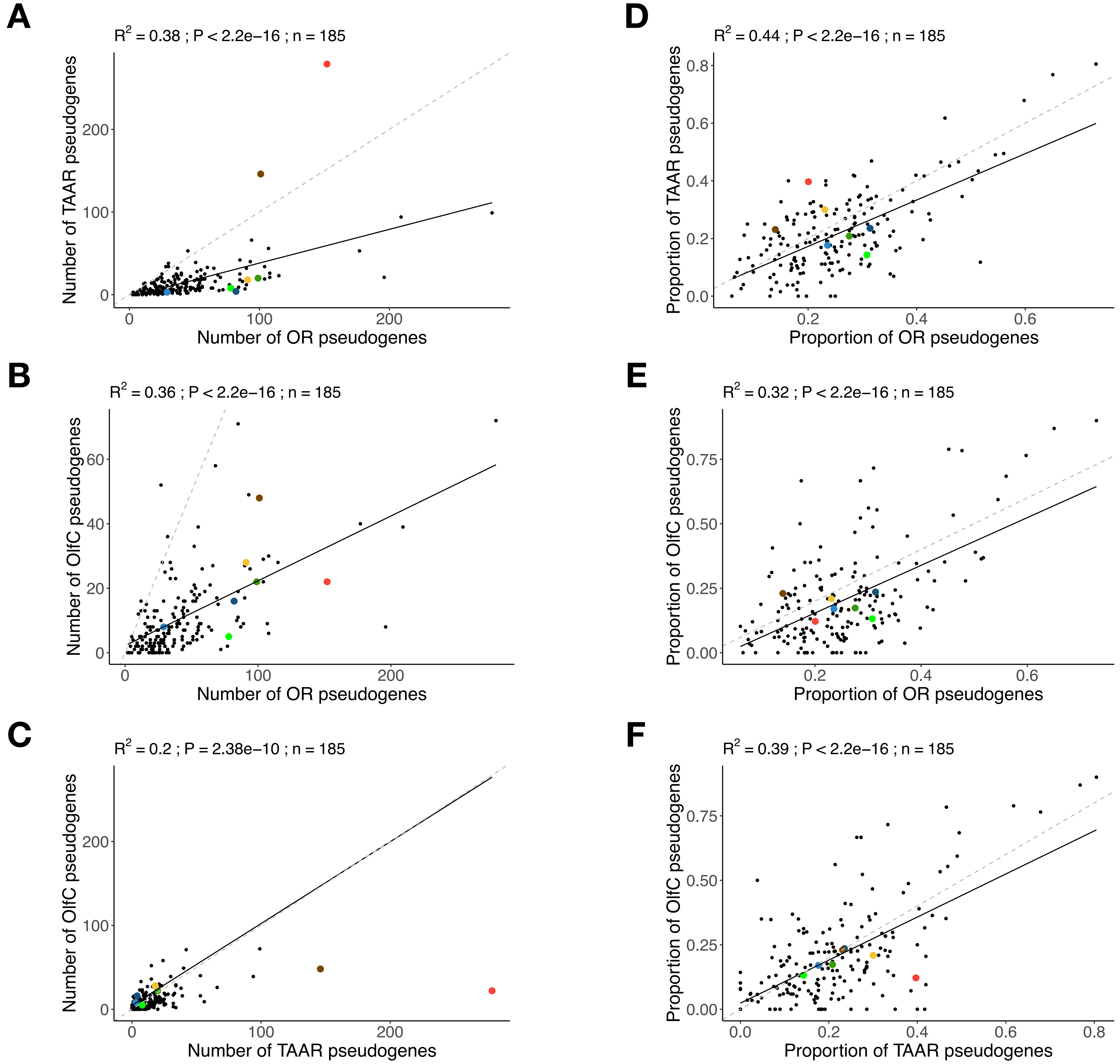
A
R^2 = 0.38 ; P < 2.2e−16 ; n = 185
Number of TAAR pseudogenes
Number of OR pseudogenes
B
R^2 = 0.36 ; P < 2.2e−16 ; n = 185
Number of OlfC pseudogenes
Number of OR pseudogenes
C
R^2 = 0.2 ; P = 2.38e−10 ; n = 185
Number of OlfC pseudogenes
Number of TAAR pseudogenes
D
R^2 = 0.44 ; P < 2.2e−16 ; n = 185
Proportion of TAAR pseudogenes
Proportion of OR pseudogenes
E
R^2 = 0.32 ; P < 2.2e−16 ; n = 185
Proportion of OlfC pseudogenes
Proportion of OR pseudogenes
F
R^2 = 0.39 ; P < 2.2e−16 ; n = 185
Proportion of OlfC pseudogenes
Proportion of TAAR pseudogenes

Fig. S5

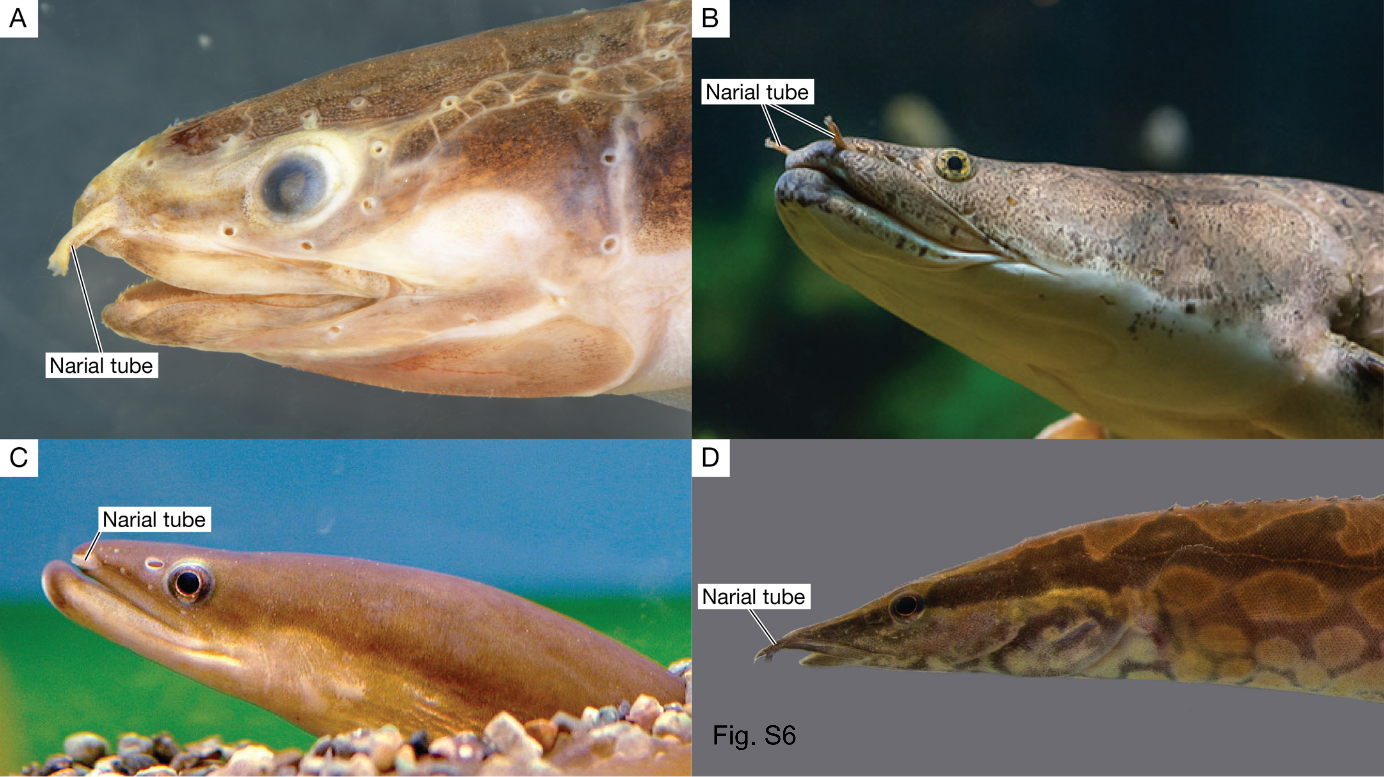

Fig. S6

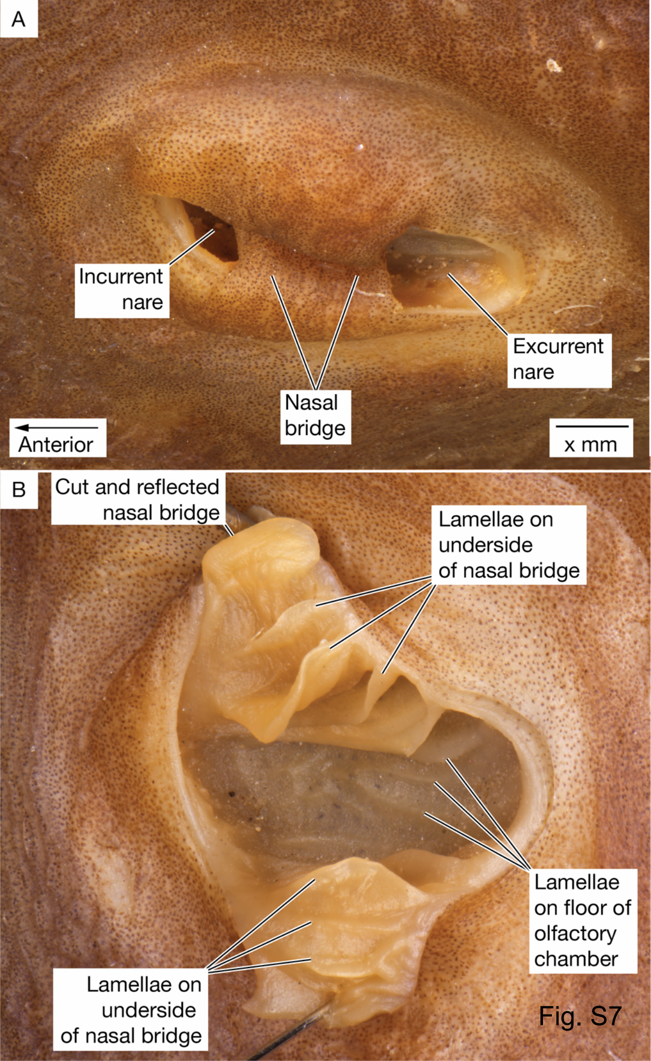
A
Incurrent nare
Nasal bridge
Excurrent nare
Anterior
x mm
B
Cut and reflected nasal bridge
Lamellae on underside of nasal bridge
Lamellae on floor of olfactory chamber
Lamellae on underside of nasal bridge
Fig. S7
