# Supplementary material for: Coevolution of the olfactory organ and its receptor repertoire in ray-finned fishes
Source: BMC Biol. 2022 Sep 1;20:195. doi: 10.1186/s12915-022-01397-x (PMC9438307; doi:10.1186/s12915-022-01397-x)

A

TAAR pipeline assessment	Present study			Previous studies			Reference
	F	I	P	F	I	P	
Danio rerio	112	1	9	109	N/A	10	Hashiguchi and Nishida 2007
Gasterosteus aculeatus	50	2	15	50	N/A	15	Azzouzi et al. 2015
Oryzias latipes	39	2	4	27	N/A	7	Azzouzi et al. 2015
Takifugu rubripes	24	0	3	13	N/A	6	Hashiguchi and Nishida 2007

B

OlfC pipeline assessment	Present study			Previous studies			Reference
	F	I	P	F	I	P	
Danio rerio	54	3	2	53	1	1	Yang et al. 2019
Gasterosteus aculeatus	22	2	2	13	1	1	Yang et al. 2019
Oryzias latipes	24	0	2	16	1	2	Yang et al. 2019
Takifugu rubripes	27	0	4	16	2	3	Yang et al. 2019

C

ORA pipeline assessment	Present study			Previous studies			Reference
	F	I	P	F	I	P	
Danio rerio	7	0	0	7	N/A	N/A	Zapilko and Korsching 2016
Gasterosteus aculeatus	5	0	1	6	N/A	N/A	Zapilko and Korsching 2016
Oryzias latipes	7	0	1	7	N/A	N/A	Zapilko and Korsching 2016
Takifugu rubripes	5	0	1	5	N/A	N/A	Zapilko and Korsching 2016

D *Danio rerio* - TAAR genes comparison

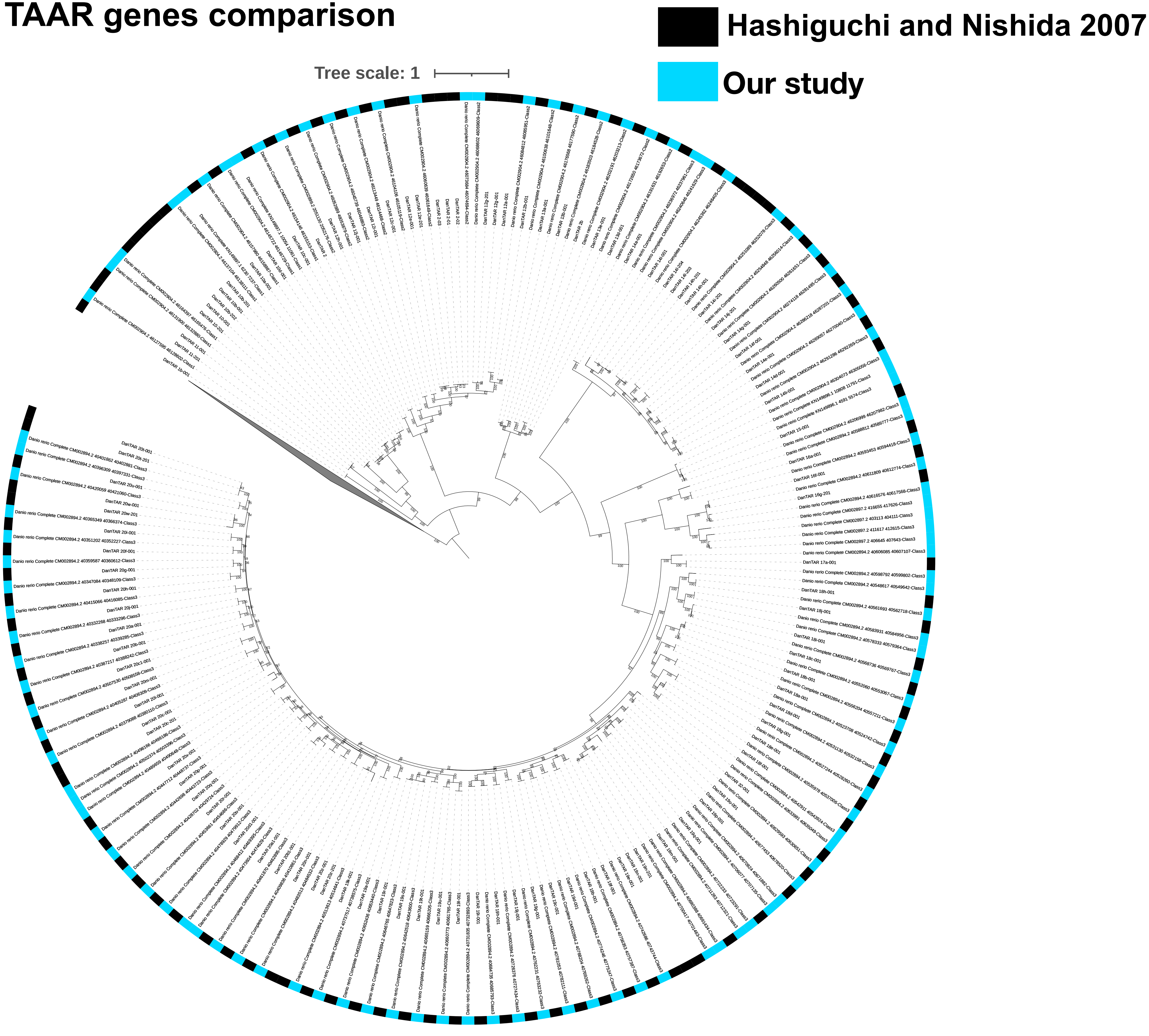

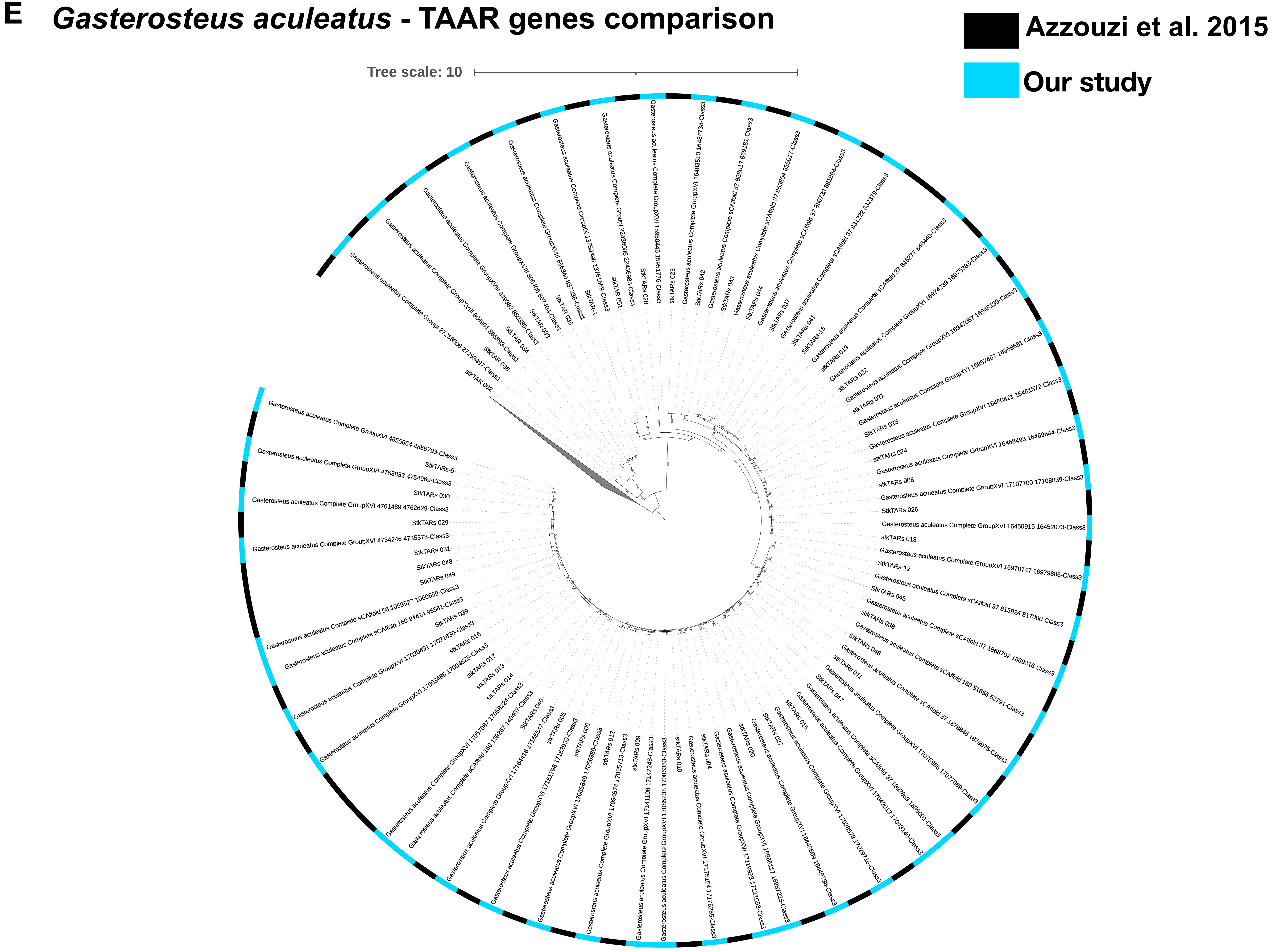
E
Gasterosteus aculeatus - TAAR genes comparison
Azzouzi et al. 2015
Our study
Tree scale: 10
Gasterosteus aculeatus Complete GroupI 27258508 27259497-Class1
stkTAR 002
Gasterosteus aculeatus Complete GroupXVIII 864901 865893-Class1
StkTAR 036
Gasterosteus aculeatus Complete GroupXVIII 849392 850380-Class1
StkTAR 034
Gasterosteus aculeatus Complete GroupXVIII 806406 807404-Class1
StkTAR 033
Gasterosteus aculeatus Complete GroupXVIII 856340 857338-Class1
StkTAR 035
Gasterosteus aculeatus Complete GroupIX 13760498 13761559-Class3
StkTAR-2
Gasterosteus aculeatus Complete GroupI 22436006 22436983-Class3
stkTAR 001
Gasterosteus aculeatus Complete GroupXVI 15950446 15951776-Class3
StkTARs 028
stkTARs 023
Gasterosteus aculeatus Complete GroupXVI 16483510 16484738-Class3
StkTARs 042
Gasterosteus aculeatus Complete sCAffold 37 868017 869181-Class3
StkTARs 043
Gasterosteus aculeatus Complete sCAffold 37 853854 855017-Class3
StkTARs 044
Gasterosteus aculeatus Complete sCAffold 37 880733 881894-Class3
StkTARs 037
Gasterosteus aculeatus Complete sCAffold 37 831222 832379-Class3
StkTARs 041
Gasterosteus aculeatus Complete sCAffold 37 845277 846440-Class3
StkTARs-15
Gasterosteus aculeatus Complete GroupXVI 16974239 16975383-Class3
stkTARs 019
Gasterosteus aculeatus Complete GroupXVI 16947057 16948199-Class3
stkTARs 022
Gasterosteus aculeatus Complete GroupXVI 16957463 16958581-Class3
stkTARs 021
Gasterosteus aculeatus Complete GroupXVI 16460421 16461572-Class3
StkTARs 025
Gasterosteus aculeatus Complete GroupXVI 16468493 16469644-Class3
stkTARs 024
stkTARs 008
Gasterosteus aculeatus Complete GroupXVI 17107700 17108839-Class3
StkTARs 026
Gasterosteus aculeatus Complete GroupXVI 16450915 16452073-Class3
stkTARs 018
Gasterosteus aculeatus Complete GroupXVI 16978747 16979886-Class3
StkTARs-12
Gasterosteus aculeatus Complete sCAffold 37 815924 817000-Class3
StkTARs 045
Gasterosteus aculeatus Complete sCAffold 37 1868702 1869816-Class3
StkTARs 038
Gasterosteus aculeatus Complete sCAffold 160 51656 52791-Class3
StkTARs 046
Gasterosteus aculeatus Complete sCAffold 37 1878846 1879975-Class3
StkTARs 011
Gasterosteus aculeatus Complete GroupXVI 17075986 17077069-Class3
StkTARs 047
Gasterosteus aculeatus Complete sCAffold 37 1893869 1895001-Class3
stkTARs 015
Gasterosteus aculeatus Complete GroupXVI 17042013 17043140-Class3
Gasterosteus aculeatus Complete GroupXVI 17028578 17029716-Class3
StkTARs 027
Gasterosteus aculeatus Complete GroupXVI 16448669 16449796-Class3
stkTARs 020
Gasterosteus aculeatus Complete GroupXVI 16966117 16967225-Class3
Gasterosteus aculeatus Complete GroupXVI 17119923 17121053-Class3
stkTARs 004
Gasterosteus aculeatus Complete GroupXVI 17175154 17176285-Class3
stkTARs 010
Gasterosteus aculeatus Complete GroupXVI 17085238 17086353-Class3
Gasterosteus aculeatus Complete GroupXVI 17141108 17142248-Class3
stkTARs 009
Gasterosteus aculeatus Complete GroupXVI 17094574 17095713-Class3
stkTARs 012
Gasterosteus aculeatus Complete GroupXVI 17065849 17066989-Class3
stkTARs 006
Gasterosteus aculeatus Complete GroupXVI 17151798 17152939-Class3
stkTARs 005
Gasterosteus aculeatus Complete GroupXVI 17164416 17165547-Class3
StkTARs 040
Gasterosteus aculeatus Complete sCAffold 160 139267 140407-Class3
Gasterosteus aculeatus Complete GroupXVI 17057087 17058224-Class3
stkTARs 014
stkTARs 013
Gasterosteus aculeatus Complete GroupXVI 17003486 17004625-Class3
stkTARs 017
Gasterosteus aculeatus Complete GroupXVI 17020491 17021630-Class3
stkTARs 016
StkTARs 039
Gasterosteus aculeatus Complete sCAffold 160 94424 95561-Class3
Gasterosteus aculeatus Complete sCAffold 56 1059527 1060659-Class3
StkTARs 049
StkTARs 048
StkTARs 031
Gasterosteus aculeatus Complete GroupXVI 4734246 4735378-Class3
StkTARs 029
Gasterosteus aculeatus Complete GroupXVI 4761489 4762629-Class3
StkTARs 030
Gasterosteus aculeatus Complete GroupXVI 4753832 4754969-Class3
StkTARs-5
Gasterosteus aculeatus Complete GroupXVI 4855664 4856793-Class3

F ***Oryzias latipes*** **- TAAR genes comparison**

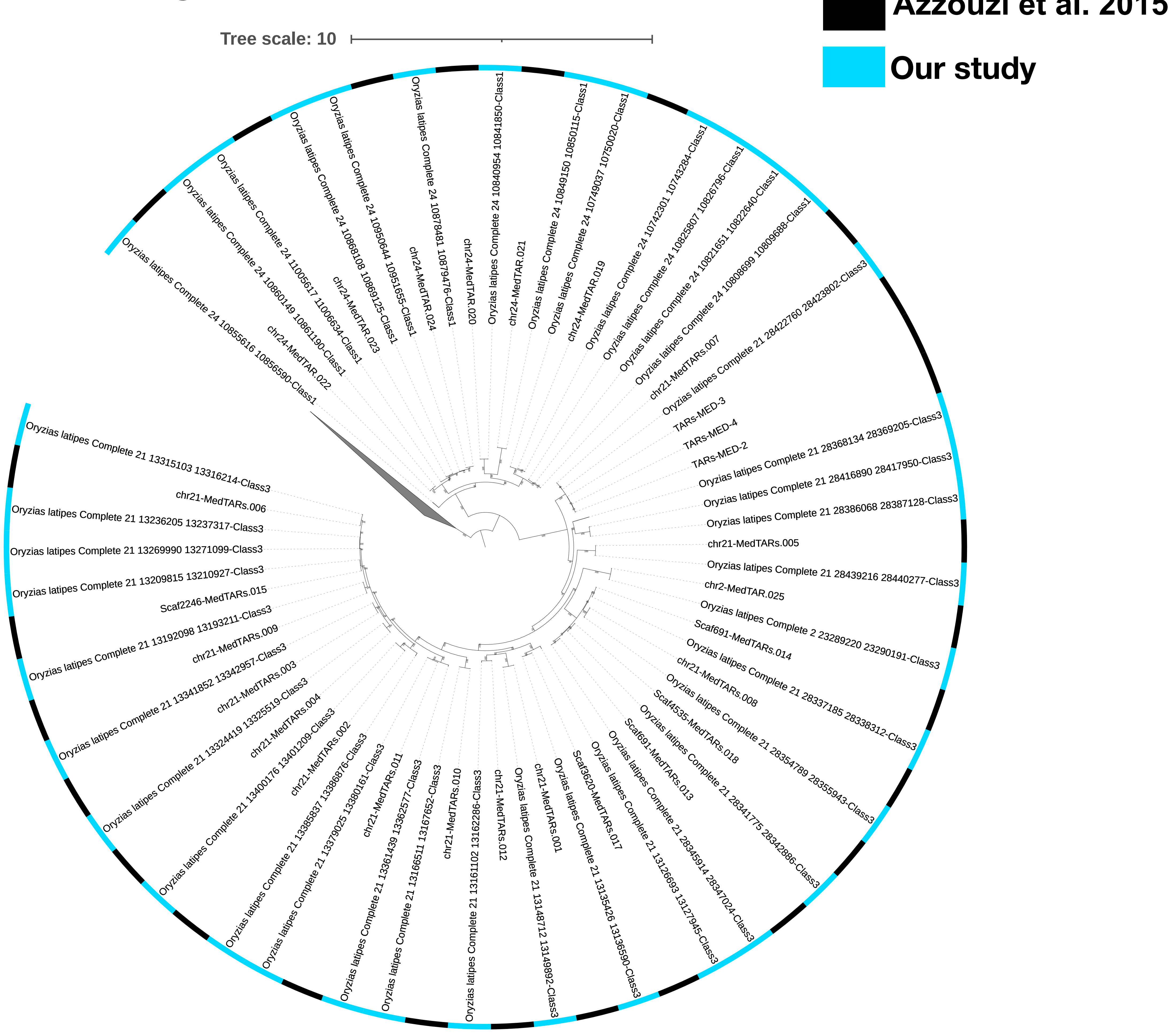

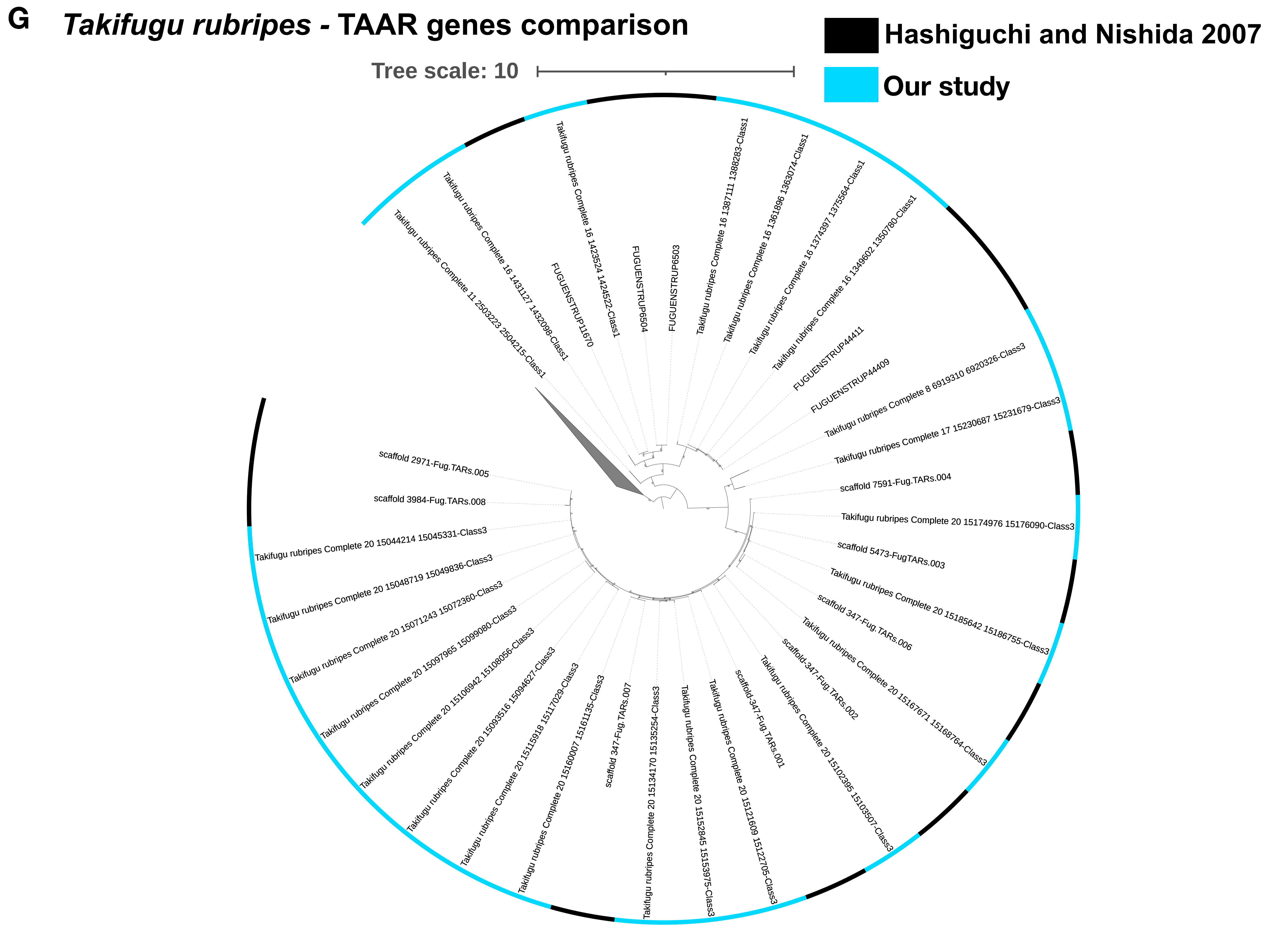

G
Takifugu rubripes - TAAR genes comparison
Hashiguchi and Nishida 2007
Our study
Tree scale: 10
Takifugu rubripes Complete 11 2503223 2504215-Class1
Takifugu rubripes Complete 16 1431127 1432098-Class1
FUGUENSTRUP11670
Takifugu rubripes Complete 16 1423524 1424522-Class1
FUGUENSTRUP6504
FUGUENSTRUP6503
Takifugu rubripes Complete 16 1387111 1388283-Class1
Takifugu rubripes Complete 16 1361896 1363074-Class1
Takifugu rubripes Complete 16 1374397 1375564-Class1
Takifugu rubripes Complete 16 1349602 1350780-Class1
FUGUENSTRUP44411
FUGUENSTRUP44409
Takifugu rubripes Complete 8 6919310 6920326-Class3
Takifugu rubripes Complete 17 15230687 15231679-Class3
scaffold 7591-Fug.TARs.004
Takifugu rubripes Complete 20 15174976 15176090-Class3
scaffold 5473-FugTARs.003
Takifugu rubripes Complete 20 15185642 15186755-Class3
scaffold 347-Fug.TARs.006
Takifugu rubripes Complete 20 15167671 15168764-Class3
scaffold-347-Fug.TARs.002
Takifugu rubripes Complete 20 15102395 15103507-Class3
scaffold-347-Fug.TARs.001
Takifugu rubripes Complete 20 15121609 15122705-Class3
Takifugu rubripes Complete 20 15152845 15153975-Class3
Takifugu rubripes Complete 20 15134170 15135254-Class3
scaffold 347-Fug.TARs.007
Takifugu rubripes Complete 20 15160007 15161135-Class3
Takifugu rubripes Complete 20 15115918 15117029-Class3
Takifugu rubripes Complete 20 15093516 15094627-Class3
Takifugu rubripes Complete 20 15106942 15108056-Class3
Takifugu rubripes Complete 20 15097965 15099080-Class3
Takifugu rubripes Complete 20 15071243 15072360-Class3
Takifugu rubripes Complete 20 15048719 15049836-Class3
Takifugu rubripes Complete 20 15044214 15045331-Class3
scaffold 3984-Fug.TARs.008
scaffold 2971-Fug.TARs.005

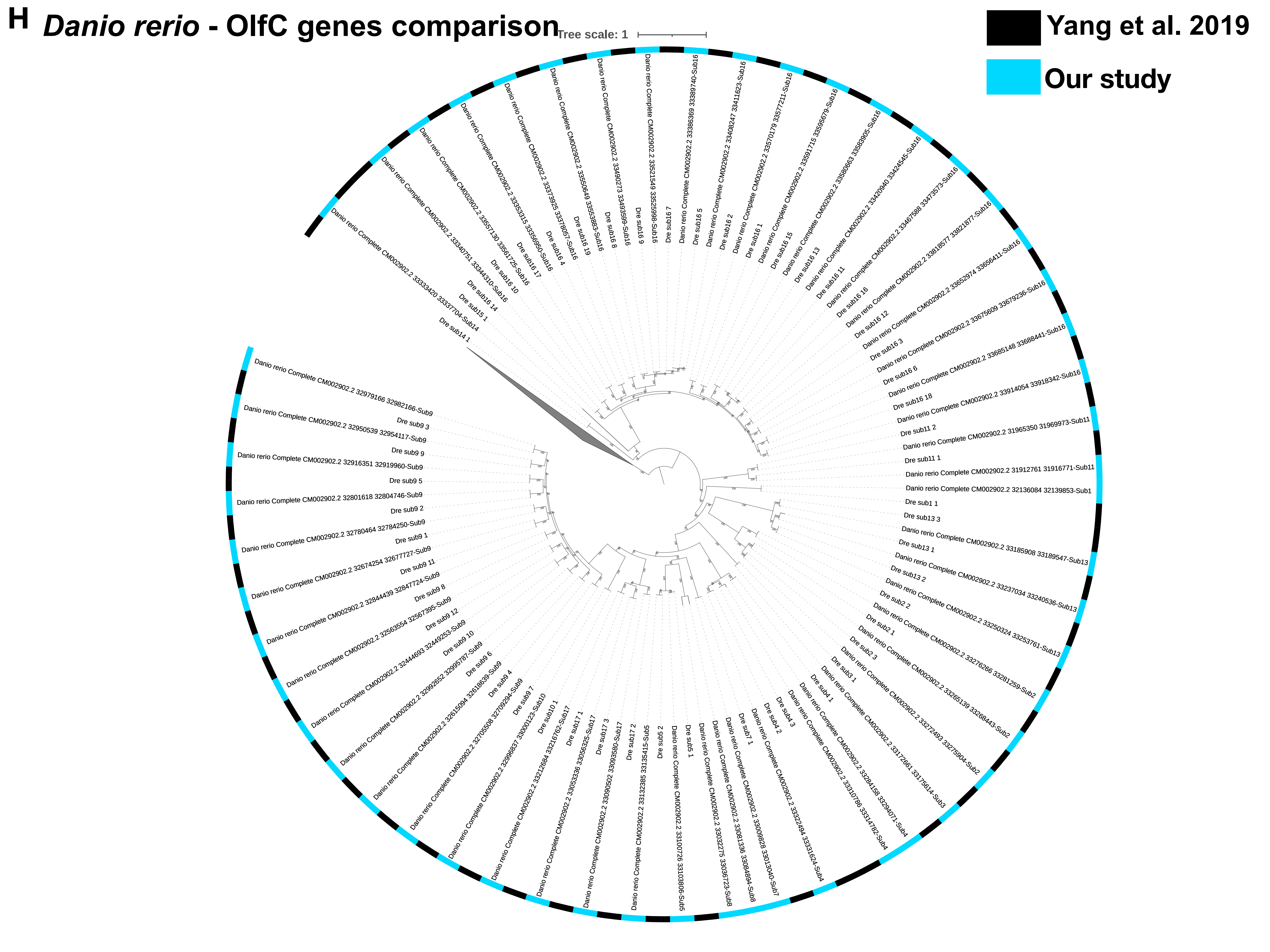
H
Danio rerio - OlfC genes comparison
Tree scale: 1
Yang et al. 2019
Our study
Dre sub14 1
Danio rerio Complete CM002902.2 33333420 33337704-Sub14
Dre sub15 1
Dre sub16 14
Danio rerio Complete CM002902.2 33340751 33344310-Sub16
Dre sub16 10
Danio rerio Complete CM002902.2 33557130 33561725-Sub16
Dre sub16 17
Danio rerio Complete CM002902.2 33353315 33356950-Sub16
Dre sub16 4
Danio rerio Complete CM002902.2 33373925 33378057-Sub16
Dre sub16 19
Danio rerio Complete CM002902.2 33550649 33553883-Sub16
Dre sub16 8
Danio rerio Complete CM002902.2 33490273 33493599-Sub16
Dre sub16 9
Danio rerio Complete CM002902.2 33521549 33525998-Sub16
Dre sub16 7
Danio rerio Complete CM002902.2 33386369 33389740-Sub16
Dre sub16 5
Dre sub16 2
Danio rerio Complete CM002902.2 33408247 33411623-Sub16
Dre sub16 1
Danio rerio Complete CM002902.2 33570179 33577211-Sub16
Dre sub16 15
Danio rerio Complete CM002902.2 33591715 33595679-Sub16
Dre sub16 13
Danio rerio Complete CM002902.2 33580663 33583905-Sub16
Dre sub16 11
Danio rerio Complete CM002902.2 33420940 33424545-Sub16
Dre sub16 16
Danio rerio Complete CM002902.2 33467588 33473573-Sub16
Dre sub16 12
Danio rerio Complete CM002902.2 33818577 33821877-Sub16
Dre sub16 3
Danio rerio Complete CM002902.2 33652974 33656411-Sub16
Dre sub16 6
Danio rerio Complete CM002902.2 33675609 33679236-Sub16
Dre sub16 18
Danio rerio Complete CM002902.2 33685148 33688441-Sub16
Danio rerio Complete CM002902.2 33914054 33918342-Sub16
Dre sub11 2
Danio rerio Complete CM002902.2 31965350 31969973-Sub11
Dre sub11 1
Danio rerio Complete CM002902.2 31912761 31916771-Sub11
Danio rerio Complete CM002902.2 32136084 32139853-Sub1
Dre sub1 1
Dre sub13 3
Danio rerio Complete CM002902.2 33185908 33189547-Sub13
Dre sub13 1
Danio rerio Complete CM002902.2 33237034 33240536-Sub13
Dre sub13 2
Danio rerio Complete CM002902.2 33250324 33253761-Sub13
Dre sub2 2
Danio rerio Complete CM002902.2 33276266 33281259-Sub2
Dre sub2 1
Dre sub2 3
Danio rerio Complete CM002902.2 33265139 33268443-Sub2
Dre sub3 1
Danio rerio Complete CM002902.2 33272493 33275904-Sub2
Dre sub4 1
Danio rerio Complete CM002902.2 33172661 33175614-Sub3
Danio rerio Complete CM002902.2 33284158 33294071-Sub4
Danio rerio Complete CM002902.2 33310786 33314782-Sub4
Dre sub4 3
Dre sub4 2
Danio rerio Complete CM002902.2 33322494 33331624-Sub4
Danio rerio Complete CM002902.2 33009828 33013040-Sub7
Dre sub7 1
Danio rerio Complete CM002902.2 33081336 33084894-Sub8
Danio rerio Complete CM002902.2 33032275 33036723-Sub8
Dre sub5 1
Danio rerio Complete CM002902.2 33100726 33103808-Sub5
Dre sub5 2
Danio rerio Complete CM002902.2 33132385 33135415-Sub5
Dre sub17 2
Danio rerio Complete CM002902.2 33090502 33093580-Sub17
Dre sub17 3
Danio rerio Complete CM002902.2 33053336 33056325-Sub17
Dre sub17 1
Danio rerio Complete CM002902.2 33212684 33216762-Sub17
Dre sub10 1
Danio rerio Complete CM002902.2 32996837 33000123-Sub10
Dre sub9 7
Danio rerio Complete CM002902.2 32705508 32709294-Sub9
Dre sub9 4
Danio rerio Complete CM002902.2 32615094 32618639-Sub9
Dre sub9 6
Danio rerio Complete CM002902.2 32992652 32995787-Sub9
Dre sub9 10
Danio rerio Complete CM002902.2 32444693 32449253-Sub9
Dre sub9 12
Danio rerio Complete CM002902.2 32563554 32567395-Sub9
Dre sub9 8
Danio rerio Complete CM002902.2 32844439 32847724-Sub9
Dre sub9 11
Danio rerio Complete CM002902.2 32674254 32677727-Sub9
Dre sub9 1
Danio rerio Complete CM002902.2 32780464 32784250-Sub9
Dre sub9 2
Danio rerio Complete CM002902.2 32801618 32804746-Sub9
Dre sub9 5
Danio rerio Complete CM002902.2 32916351 32919960-Sub9
Dre sub9 9
Danio rerio Complete CM002902.2 32950539 32954117-Sub9
Dre sub9 3
Danio rerio Complete CM002902.2 32979166 32982166-Sub9

I ***Gasterosteus aculeatus*** **- OlfC genes comparison**

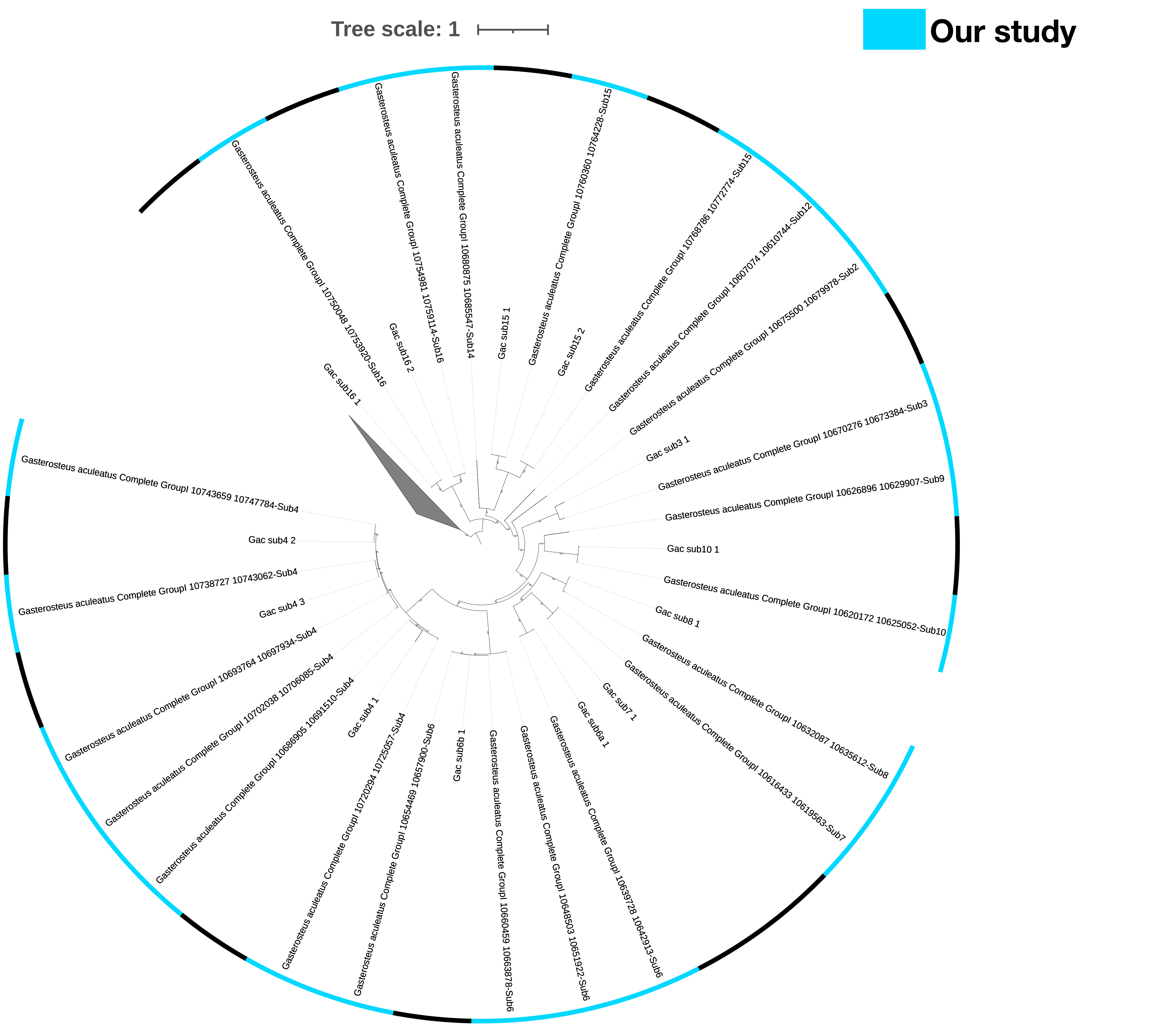

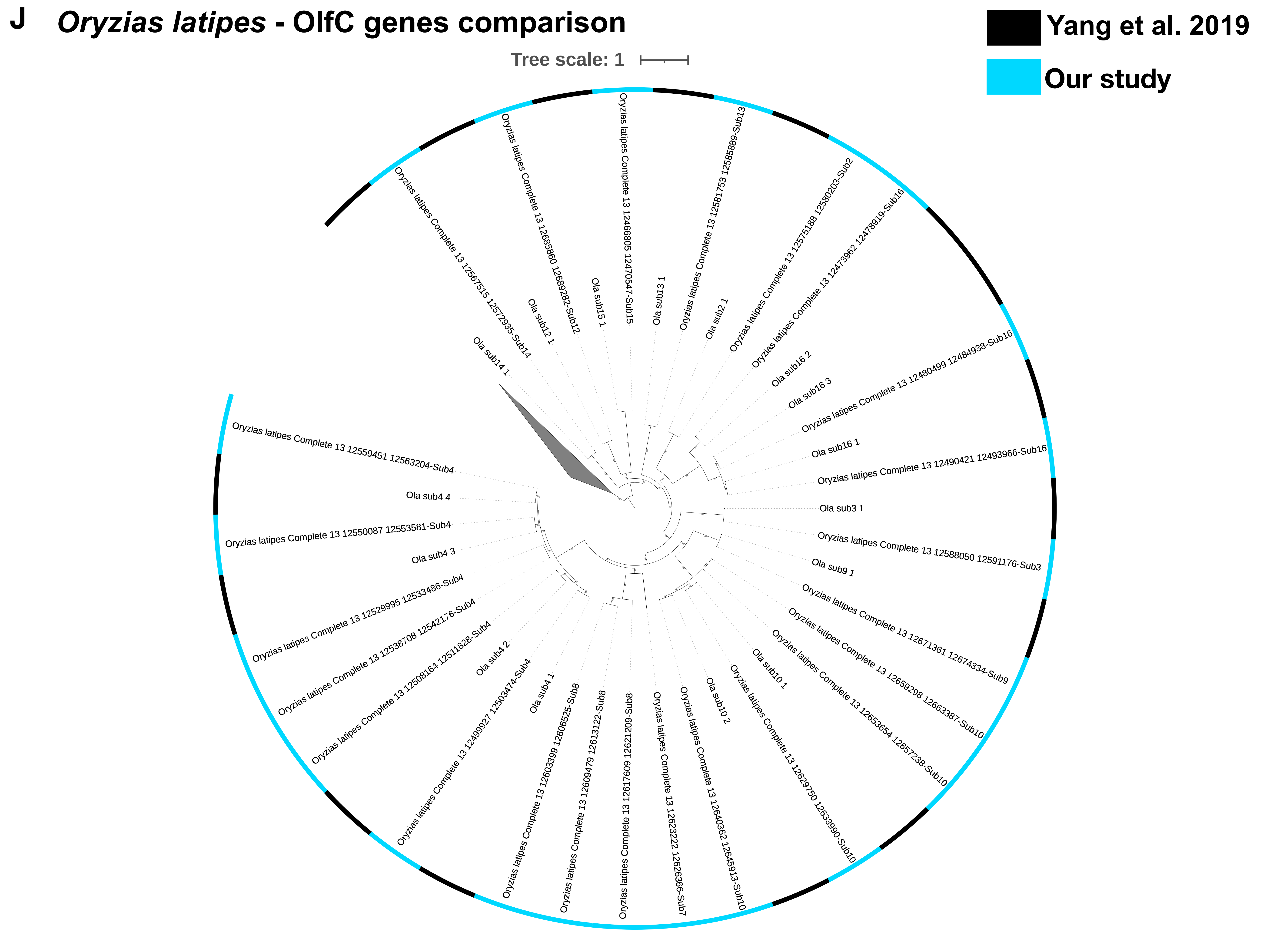

J
Oryzias latipes - OlfC genes comparison
Tree scale: 1
Yang et al. 2019
Our study
Oryzias latipes Complete 13 12559451 12563204-Sub4
Ola sub4 4
Oryzias latipes Complete 13 12550087 12553581-Sub4
Ola sub4 3
Oryzias latipes Complete 13 12529995 12533486-Sub4
Oryzias latipes Complete 13 12538708 12542176-Sub4
Oryzias latipes Complete 13 12508164 12511828-Sub4
Ola sub4 2
Oryzias latipes Complete 13 12499927 12503474-Sub4
Ola sub4 1
Oryzias latipes Complete 13 12603399 12606525-Sub8
Oryzias latipes Complete 13 12609479 12613122-Sub8
Oryzias latipes Complete 13 12617609 12621209-Sub8
Oryzias latipes Complete 13 12623222 12626366-Sub7
Oryzias latipes Complete 13 12640362 12645913-Sub10
Ola sub10 2
Oryzias latipes Complete 13 12629750 12633990-Sub10
Ola sub10 1
Oryzias latipes Complete 13 12653654 12657238-Sub10
Oryzias latipes Complete 13 12659298 12663387-Sub10
Oryzias latipes Complete 13 12671361 12674334-Sub9
Ola sub9 1
Oryzias latipes Complete 13 12588050 12591176-Sub3
Ola sub3 1
Oryzias latipes Complete 13 12490421 12493966-Sub16
Ola sub16 1
Oryzias latipes Complete 13 12480499 12484938-Sub16
Ola sub16 3
Ola sub16 2
Oryzias latipes Complete 13 12473962 12478919-Sub16
Oryzias latipes Complete 13 12575188 12580203-Sub2
Ola sub2 1
Oryzias latipes Complete 13 12581753 12585889-Sub13
Ola sub13 1
Oryzias latipes Complete 13 12466805 12470547-Sub15
Ola sub15 1
Oryzias latipes Complete 13 12685860 12689282-Sub12
Ola sub12 1
Oryzias latipes Complete 13 12567515 12572935-Sub14
Ola sub14 1

K ***Takifugu rubripes*** **- OlfC genes comparison**

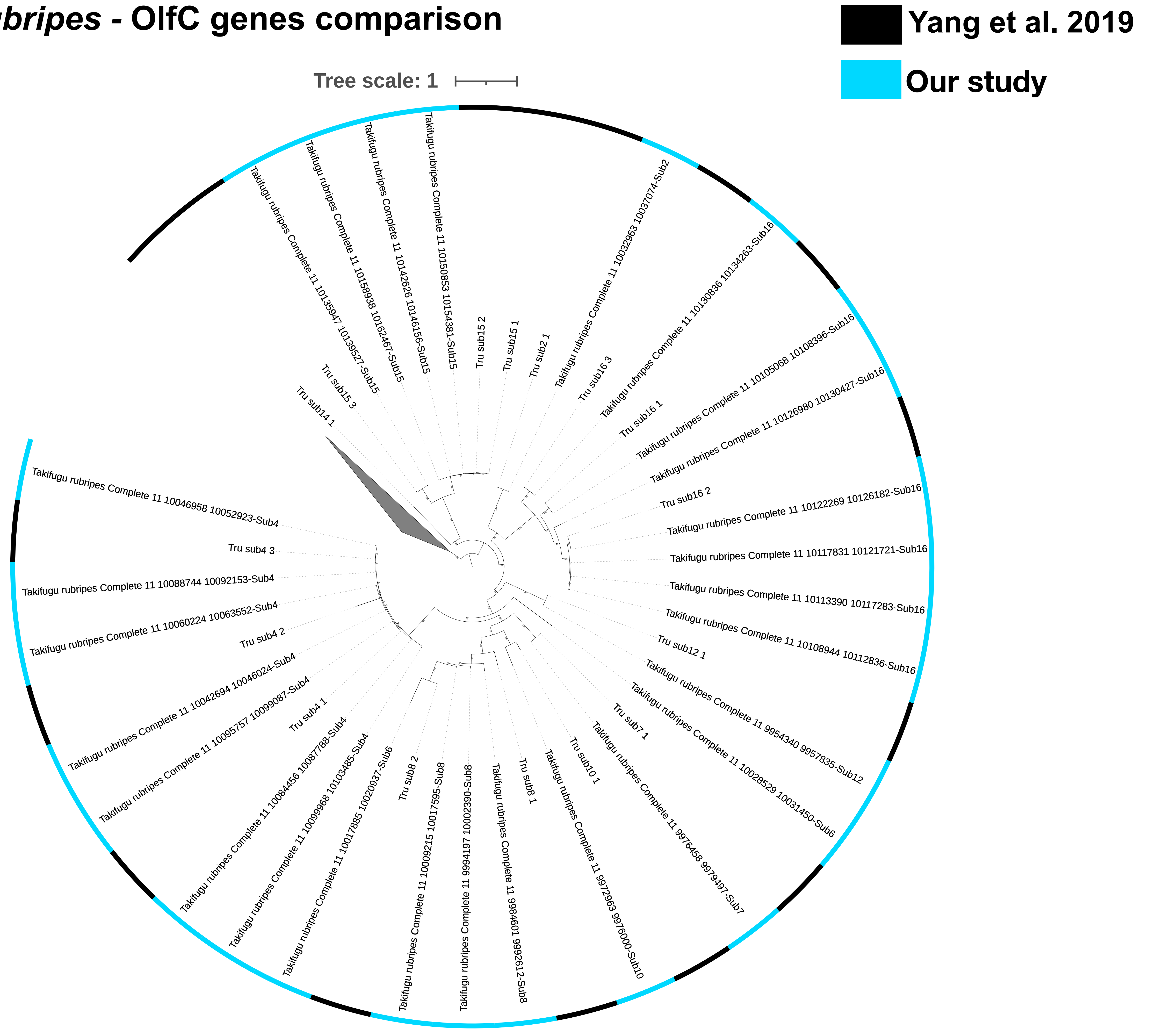

L

Danio rerio - ORA genes comparison

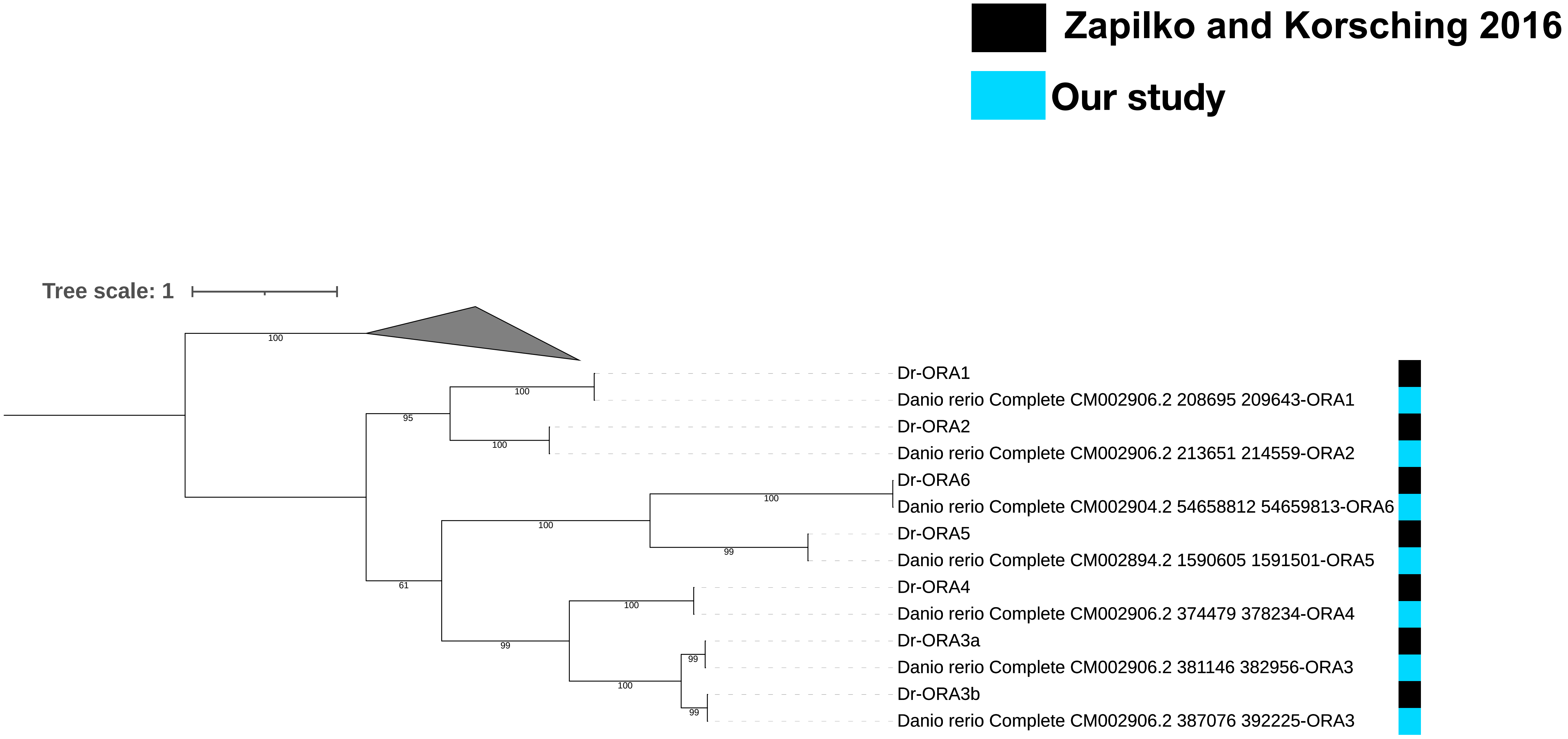

M ***Gasterosteus aculeatus* - ORA genes comparison**

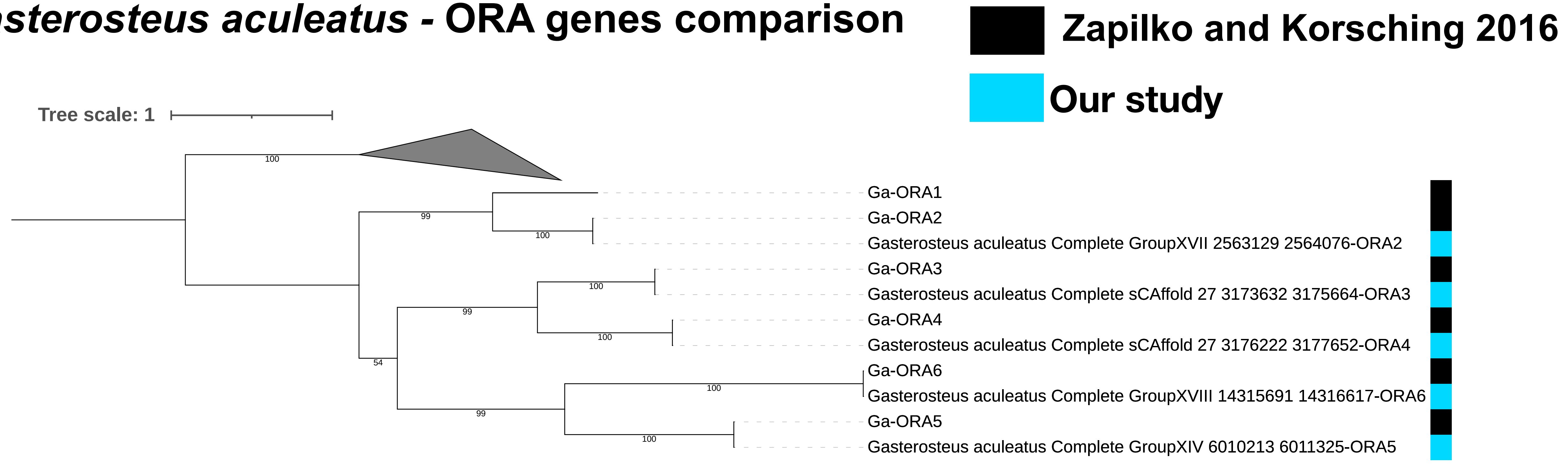

ORA1 is found as pseudogene (insertion of 2bp) in the current stickleback genome assembly (BROADS1)

```
C4 Alignment:
------------
         Query: Ga-ORA1
        Target: GroupXVII:2554871-2563367 [revcomp]
         Model: protein2genome:bestfit
     Raw score: 1276
   Query range: 0 -> 313
  Target range: 4958 -> 4001

    1 : MetAspLeuCysValThrIleLysGlyValSerPheLeuLeuGlnThrGlyMetGlyIleLe :   21
        ||||||||||||||||||||||||||||||||||||||||||||||||||||||||||||||
        MetAspLeuCysValThrIleLysGlyValSerPheLeuLeuGlnThrGlyMetGlyIleLe
 4958 : ATGGATCTGTGCGTCACCATCAAAGGGGTCTCCTTCCTCCTGCAAACAGGCATGGGCATCTT : 4898

   22 : uGlyAsnThrValValLeuLeuAlaTyrAlaGlnLeuIleTyrAlaGluProLysLeuLeuP :   42
        ||||||||||||||||||||||||||||||||||||||||||||||||||||||||||||||
        uGlyAsnThrValValLeuLeuAlaTyrAlaGlnLeuIleTyrAlaGluProLysLeuLeuP
 4897 : AGGGAACACGGTGGTGCTGCTGGCCTACGCTCAGCTCATCTACGCCGAGCCCAAGCTCCTAC : 4835

   43 : roValAspMetIleLeuCysHisLeuAlaPheAlaAsnLeuMetLeuLeuLeuThrArgCys :   62
        ||||||||||||||||||||||||||||||||||||||||||||||||||||||||||||||
        roValAspMetIleLeuCysHisLeuAlaPheAlaAsnLeuMetLeuLeuLeuThrArgCys
 4834 : CCGTGGACATGATCCTGTGCCACCTGGCCTTCGCCAACCTGATGCTGCTGCTGACCCGCTGC : 4775

   63 : ValProGlnThrMetSerValPheGlyLeuArgAspLeuLeuGlyAspProGlyCysLysVa :   83
        ||||||||||||||||||||||||||||||||||||||||||||||||||||||||||||||
        ValProGlnThrMetSerValPheGlyLeuArgAspLeuLeuGlyAspProGlyCysLysVa
 4774 : GTCCCGCAGACCATGAGCGTGTTCGGGCTGAGGGACCTGCTGGGTGACCCCGGCTGCAAGGT : 4712

   84 : lValIleTyrAlaTyrArgIleGlyArgAlaLeuSerValCysValThrCysMetLeuSerV :  104
        ||||||||||||||||||||||||||||||||||||||||||||||||||||||||||||||
        lValIleTyrAlaTyrArgIleGlyArgAlaLeuSerValCysValThrCysMetLeuSerV
 4711 : GGTGATCTACGCCTACCGCATCGGCCGGGCTTTGTCGGTCTGCGTCACCTGCATGCTCAGCG : 4649

  105 : alPheGlnAlaValThrLeuAlaPro--AlaGlyProArgLeuSerArgLeuLysProAlaL :  124
        ||||||||||||||||||||   |||##||||||||||||||||||||||||||||||||||
        alPheGlnAlaValThrLeu---Pro##AlaGlyProArgLeuSerArgLeuLysProAlaL
 4648 : TCTTTCAGGCGGTGACCTTG---CCCTCGCCGGACCCCGTCTGTCACGGTTGAAGCCCGCAC : 4590
```

N

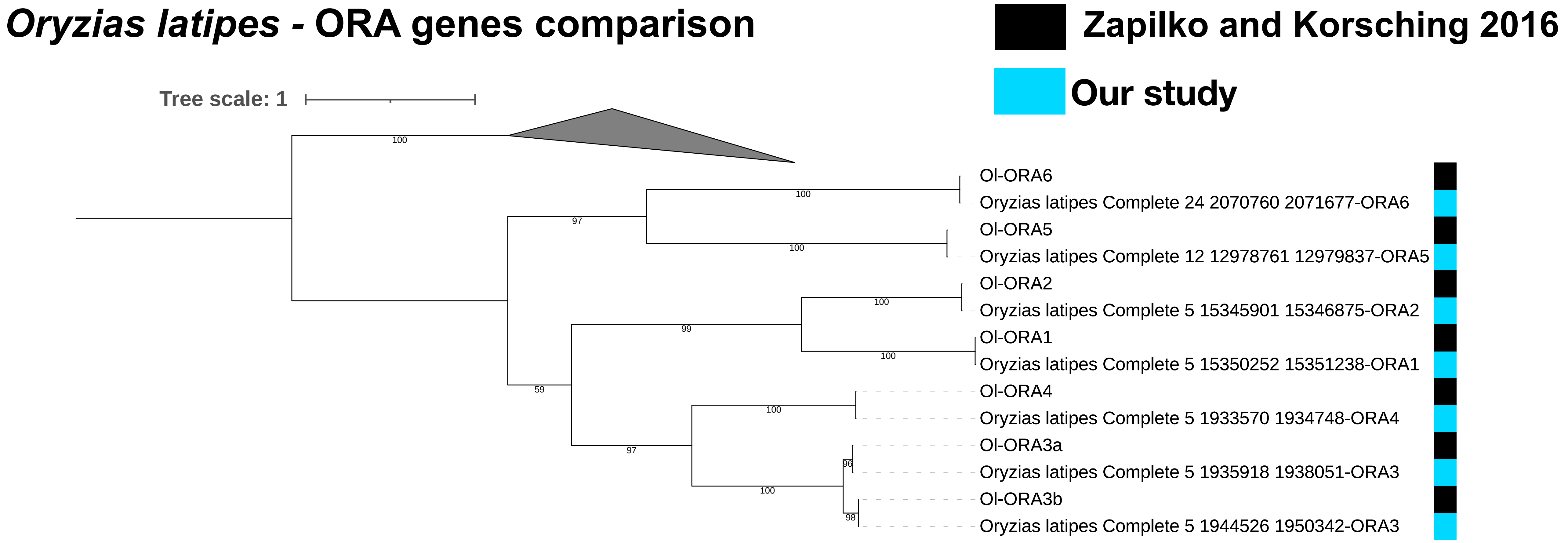

Oryzias latipes - ORA genes comparison
Zapilko and Korsching 2016
Our study
Tree scale: 1
Ol-ORA6
Oryzias latipes Complete 24 2070760 2071677-ORA6
Ol-ORA5
Oryzias latipes Complete 12 12978761 12979837-ORA5
Ol-ORA2
Oryzias latipes Complete 5 15345901 15346875-ORA2
Ol-ORA1
Oryzias latipes Complete 5 15350252 15351238-ORA1
Ol-ORA4
Oryzias latipes Complete 5 1933570 1934748-ORA4
Ol-ORA3a
Oryzias latipes Complete 5 1935918 1938051-ORA3
Ol-ORA3b
Oryzias latipes Complete 5 1944526 1950342-ORA3

O

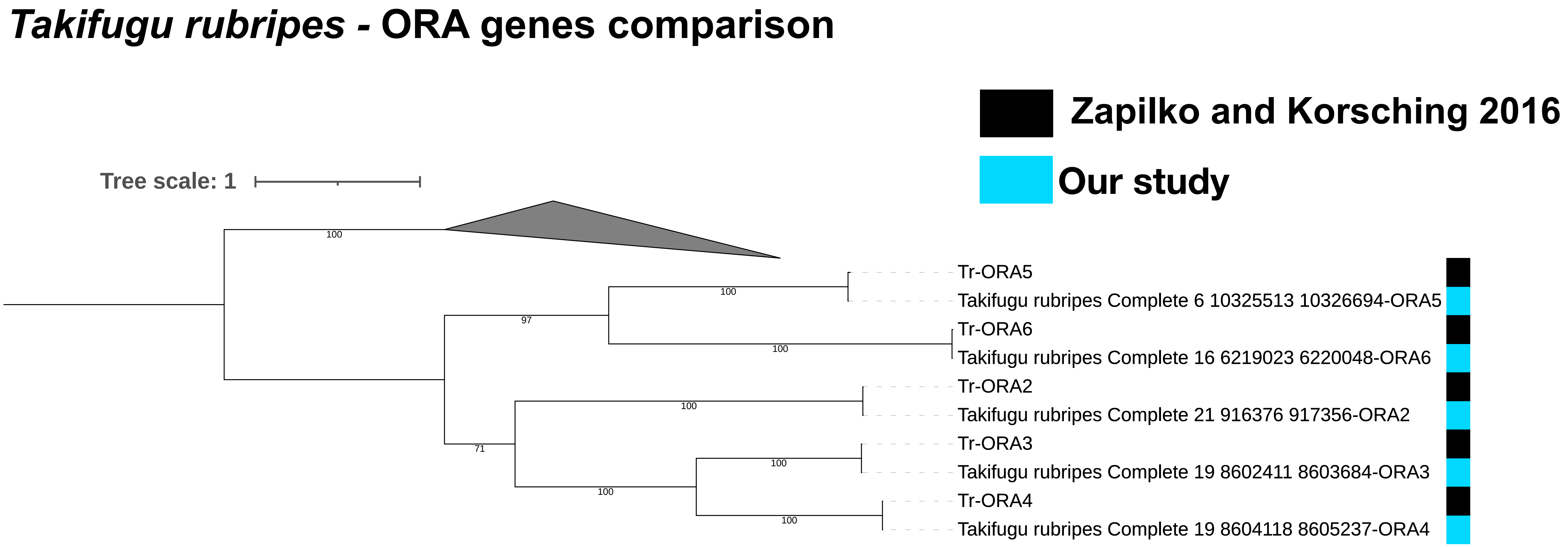

Takifugu rubripes - ORA genes comparison
Zapilko and Korsching 2016
Our study
Tree scale: 1
Tr-ORA5
Takifugu rubripes Complete 6 10325513 10326694-ORA5
Tr-ORA6
Takifugu rubripes Complete 16 6219023 6220048-ORA6
Tr-ORA2
Takifugu rubripes Complete 21 916376 917356-ORA2
Tr-ORA3
Takifugu rubripes Complete 19 8602411 8603684-ORA3
Tr-ORA4
Takifugu rubripes Complete 19 8604118 8605237-ORA4

P ***Polypterus senegalus*** **- OR genes comparison**

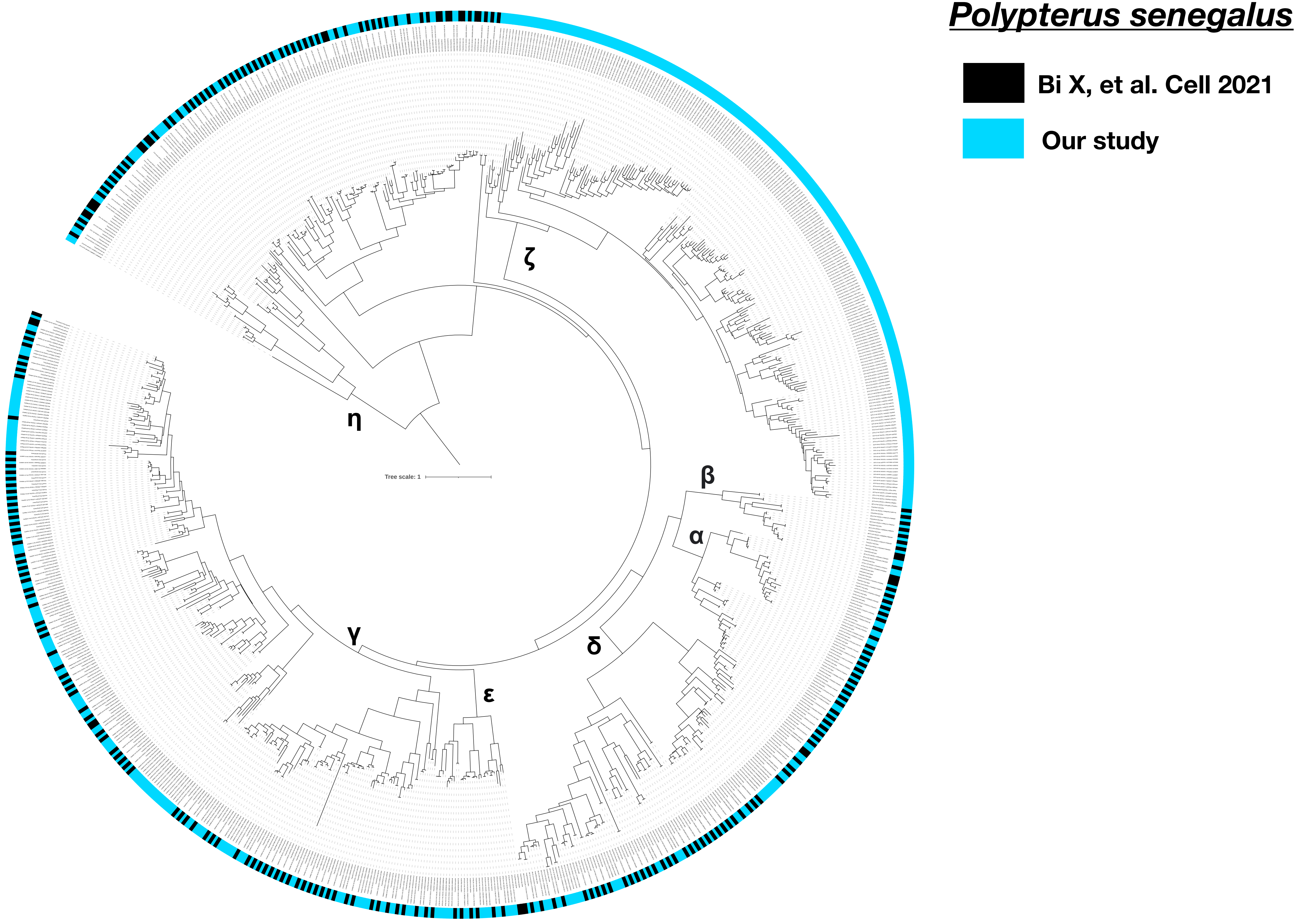

Supplement: Supplementary file 3 — Additional file 3. Supplementary Data 2. Comparison of olfactory receptor gene repertoires from the present and previous studies. (A) Summary of the number of TAAR genes retrieved in our study and previous studies of four teleost species. (B) Summary of the number of OlfC genes retrieved in our study and previous studies of four teleost species. (C) Summary of the number of ORA genes retrieved in our study and previous studies of four teleost species. (D) Phylogenetic tree of Danio rerio TAAR genes retrieved in Hashiguchi and Nishida 2007 and our study. (E) Phylogenetic tree of Gasterosteus aculeatus TAAR genes retrieved in Azzouzi et al. 2015 and our study. (F) Phylogenetic tree of Oryzias latipes TAAR genes retrieved in Azzouzi et al. 2015 and our study. (G) Phylogenetic tree of Takifugu rubripes TAAR genes retrieved in Hashiguchi and Nishida 2007 and our study. (H) Phylogenetic tree of Danio rerio OlfC genes retrieved in Yang et al. 2019 and our study. (I) Phylogenetic tree of Gasterosteus aculeatus OlfC genes retrieved in Yang et al. 2019 and our study. (J) Phylogenetic tree of Oryzias latipes OlfC genes retrieved in Yang et al. 2019 and our study. (K) Phylogenetic tree of Takifugu rubripes OlfC genes retrieved in Yang et al. 2019 and our study. (L) Phylogenetic tree of Danio rerio ORA genes retrieved in Zapilko and Korsching 2016 and our study. (M) Phylogenetic tree of Gasterosteus aculeatus ORA genes retrieved in Zapilko and Korsching 2016 and our study. (N) Phylogenetic tree of Oryzias latipes ORA genes retrieved in Zapilko and Korsching 2016 and our study. (O) Phylogenetic tree of Takifugu rubripes ORA genes retrieved in Zapilko and Korsching 2016 and our study. (P) Phylogenetic tree of Polypterus senegalus OR genes retrieved in Bi X et al. 2021 and our study. [file 12915_2022_1397_MOESM3_ESM.pdf]
