# Supplementary figures and images for: Coevolution of the olfactory organ and its receptor repertoire in ray-finned fishes
Source: BMC Biol. 2022 Sep 1;20:195. doi: 10.1186/s12915-022-01397-x (PMC9438307; doi:10.1186/s12915-022-01397-x)

(A)

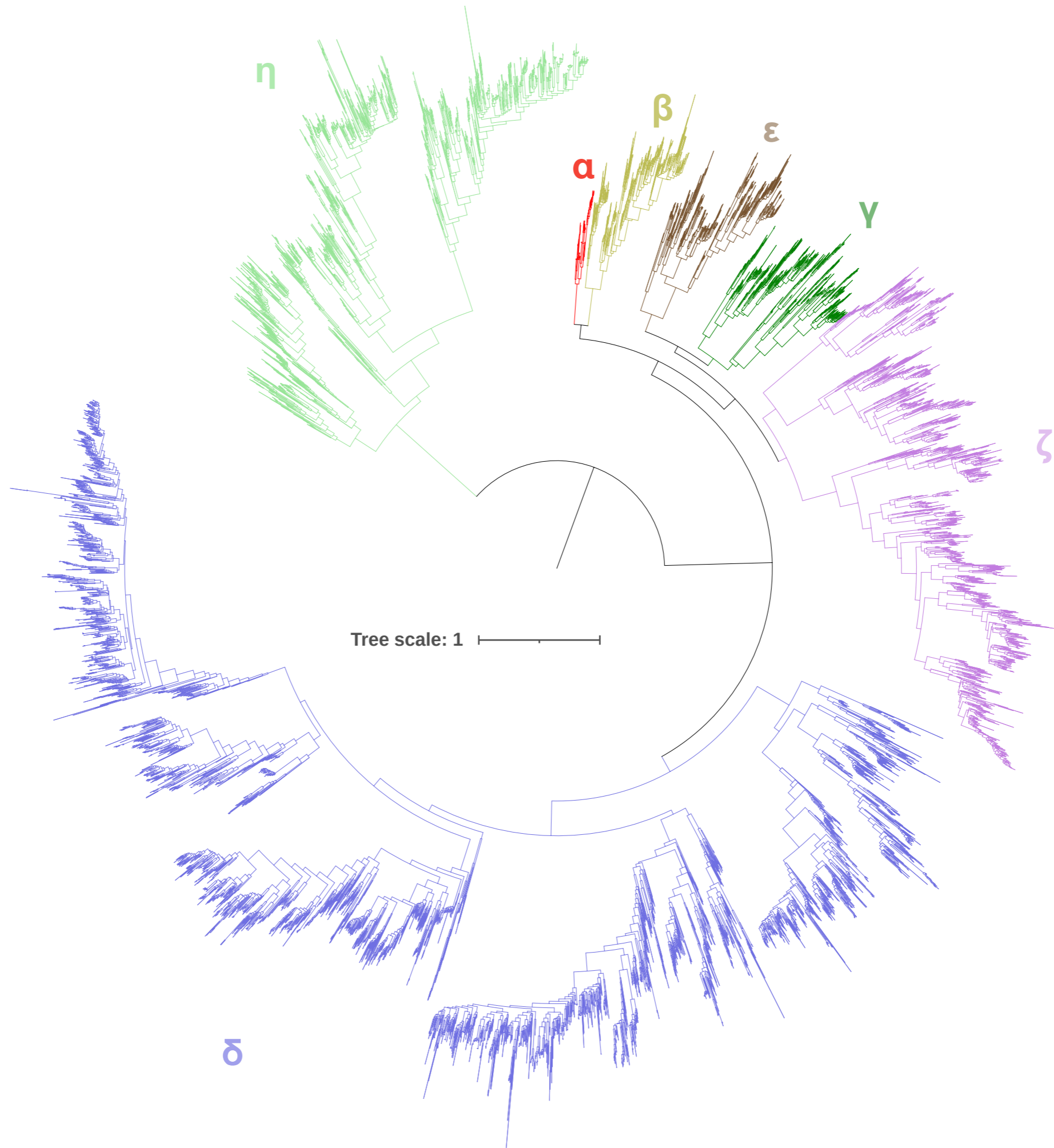

(B)

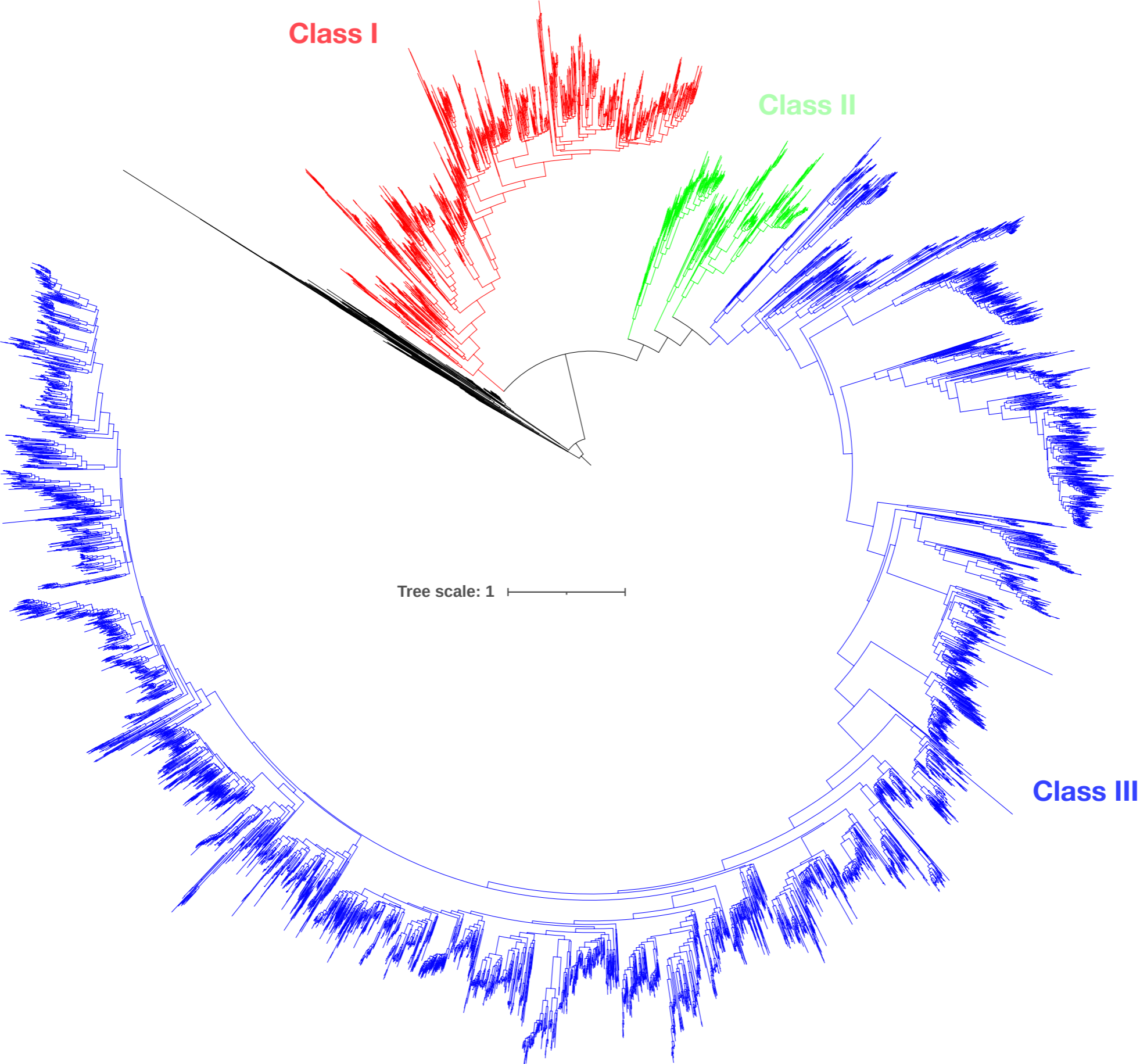

(C)

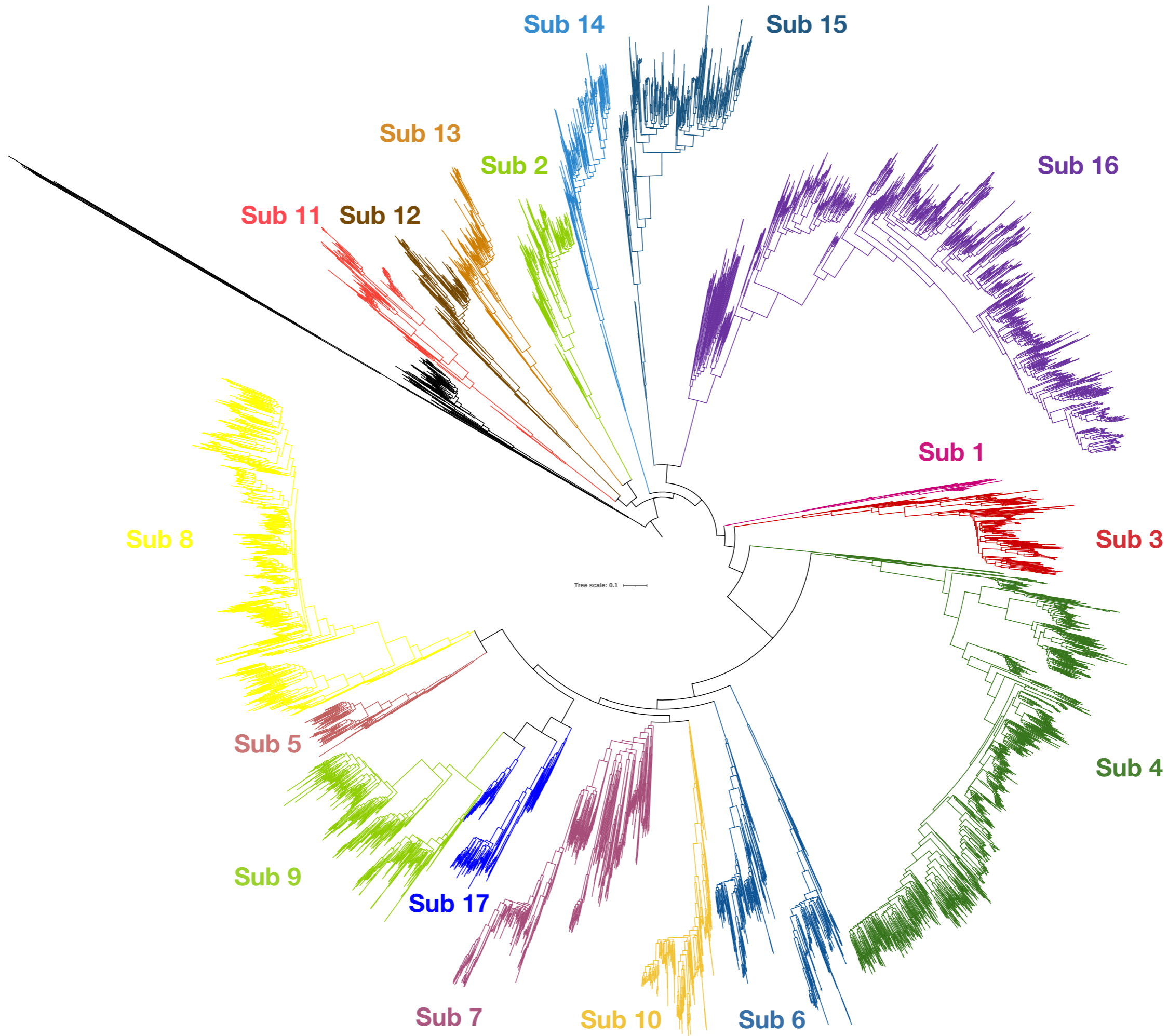

(D)

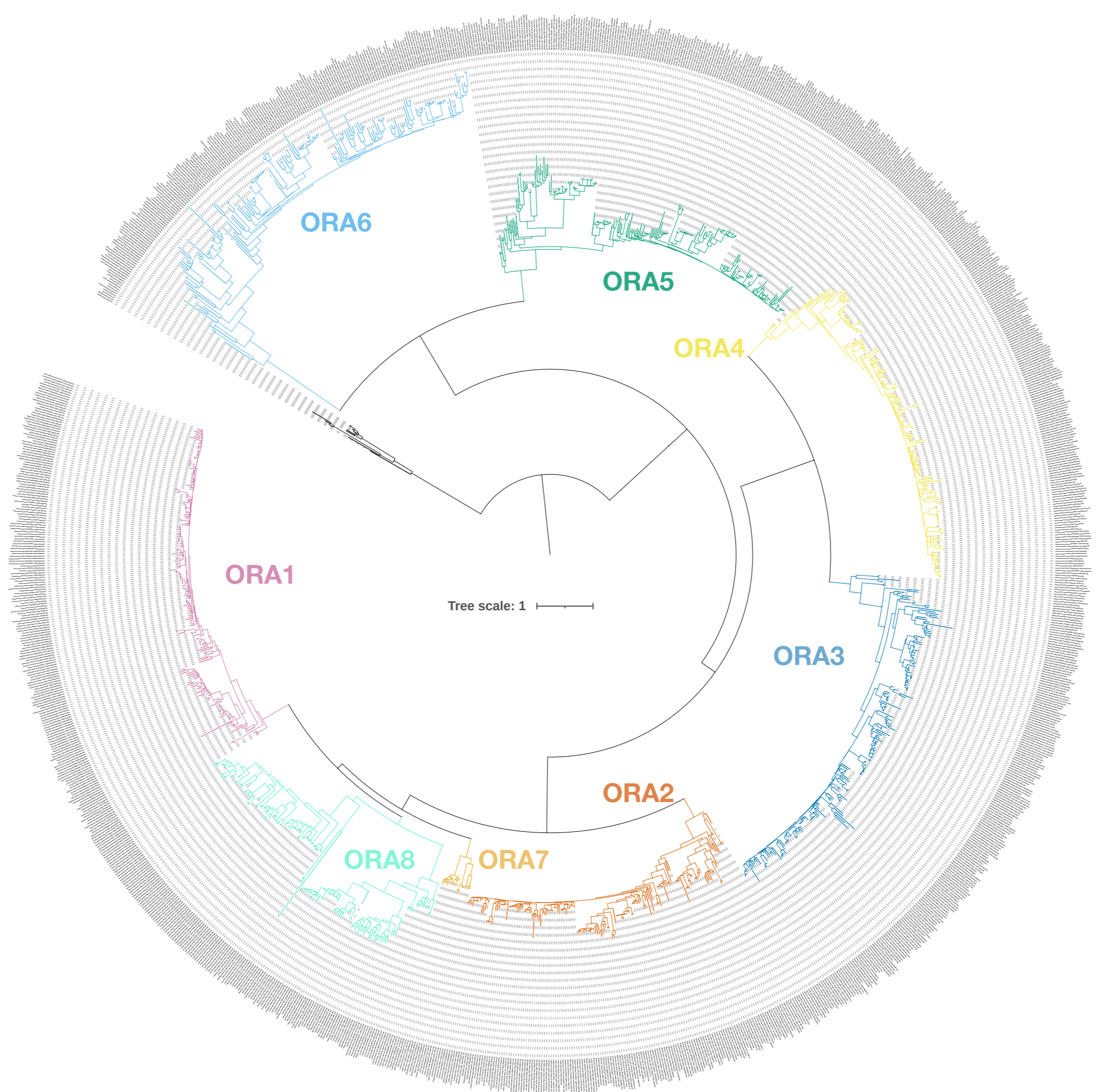

Supplement: Supplementary file 4 — Additional file 4. Supplementary Data 3. (A) Phylogeny of OR genes from 44 species representing 44 orders of ray-finned fishes sampled in this study. Branches are colored according to the gene subfamily classification. (B) Phylogeny of all TAAR genes retrieved from 185 ray-finned fishes. Branches are colored according to gene family classification. Outgroup sequences (nonTAAR GPCRs) are colored in black. (C) Phylogeny of all OlfC genes retrieved from 185 ray-finned fish. Branches are colored according to the gene subfamily classification. Outgroup sequences (CasR and V2R2 genes) are colored in black. (D) Phylogeny of all ORA genes retrieved from 185 ray-finned fishes. Branches are colored according to the gene subfamily classification. Outgroup sequences (T2R genes) are colored in black. [file 12915_2022_1397_MOESM4_ESM.pdf]
